# Reactivating Hippo by drug compounds to suppress gastric cancer and enhance chemotherapy sensitivity

**DOI:** 10.1016/j.jbc.2024.107311

**Published:** 2024-04-22

**Authors:** Zhifa Cao, Yu Hou, Zhangting Zhao, Hui Zhang, Luyang Tian, Yiming Zhang, Chao Dong, Fenghua Guo, Lijie Tan, Yi Han, Wenjia Wang, Shi Jiao, Yang Tang, Liwei An, Zhaocai Zhou

**Affiliations:** 1 State Key Laboratory of Genetic Engineering, School of Life Sciences, Zhongshan Hospital, Fudan University, Shanghai, China; 2 Department of Stomatology, Department of Medical Ultrasound, Shanghai Tenth People's Hospital, Tongji University Cancer Center, School of Medicine, Tongji University, Shanghai, China; 3 Department of Medical Oncology, The First Affiliated Hospital of Kunming Medical University, Kunming, China; 4 Department of General Surgery, Hua’shan Hospital, Fudan University Shanghai Medical College, Shanghai, China; 5 Department of Thoracic Surgery, Cancer Center, Zhongshan Hospital, Fudan University, Shanghai, China; 6 Collaborative Innovation Center for Cancer Personalized Medicine, School of Public Health, Nanjing Medical University, Nanjing, China

**Keywords:** Hippo signaling, STRIPAK, drug compound, gastric cancer, chemotherapy

## Abstract

The Hippo signaling pathway plays an essential role in organ size control and tumorigenesis. Loss of Hippo signal and hyper-activation of the downstream oncogenic YAP signaling are commonly observed in various types of cancers. We previously identified STRN3-containing PP2A phosphatase as a negative regulator of MST1/2 kinases (*i.e.*, Hippo) in gastric cancer (GC), opening the possibility of selectively targeting the PP2Aa–STRN3–MST1/2 axis to recover Hippo signaling against cancer. Here, we further discovered 1) disulfiram (DSF), an FDA-approved drug, which can similarly block the binding of STRN3 to PP2A core enzyme and 2) CX-6258 (CX), a chemical inhibitor, that can disrupt the interaction between STRN3 and MST1/2, both allowing reactivation of Hippo activity to inhibit GC. More importantly, we found these two compounds, *via* an MST1/2 kinase-dependent manner, inhibit DNA repair to sensitize GC towards chemotherapy. In addition, we identified thiram, a structural analog of DSF, can function similarly to inhibit cancer cell proliferation or enhance chemotherapy sensitivity. Interestingly, inclusion of copper ion enhanced such effects of DSF and thiram on GC treatment. Overall, this work demonstrated that pharmacological targeting of the PP2Aa–STRN3–MST1/2 axis by drug compounds can potently recover Hippo signal for tumor treatment.

The evolutionarily conserved Hippo signaling pathway plays important roles in organ size control, tissue homeostasis, and tumorigenesis (1, 2, 3). In the canonical signaling cascade of mammalian Hippo pathway, MST1/2 kinases (*i.e.*, Hippo) phosphorylate and activate LATS1/2 kinases, which in turn phosphorylate the transcriptional coactivators YAP/TAZ, causing their cytoplasmic sequestration and degradation. Once the Hippo signal is absent, YAP/TAZ will translocate into the nucleus, where they form a complex with TEAD family of transcription factors to promote the expression of downstream target genes such as *CTGF* and *CYR61*, leading to increased cell proliferation and decreased apoptosis (4, 5, 6). Accordingly, dysregulation of the Hippo-YAP signaling, for example, loss of Hippo signal and hyperactivation of YAP/TAZ are frequently observed in various types of tumors (7, 8, 9), making this pathway as an attractive drug target for cancer treatment.

Growing efforts have been made to dissect the regulatory effect of the Hippo-YAP signaling in various types of malignant tumors including gastric cancer (GC) (10, 11, 12, 13, 14). Based on the mechanistic understanding of Hippo-YAP pathological function, various therapeutic strategies have been developed. Roughly, the current Hippo-targeting strategies can be classified into two directions: either by suppressing the YAP nuclear translocation *via* enhanced Hippo kinase activity or by disrupting the nuclear YAP/TAZ–TEAD complex formation to suppress gene transcription (15). Specifically, STRN3-derived Hippo-activating peptide (SHAP) (16) and SuperHippo peptide (17) represent two kinase activators for MST1/2 and LATS1/2, respectively. Alternatively, compounds such as verteporfin, digitoxin, and flufenamic acid (18, 19, 20, 21, 22), as well as peptides including Super-TDU (23), YAP cyclic peptide (peptide 17) (24), and cystine-dense peptide (TB1G1) (25) have been identified or developed to disrupt the YAP–TEAD complex.

The striatin-interacting phosphatase and kinases (STRIPAK) complexes have been identified as a signaling hub at the upstream of the Hippo pathway (26, 27, 28, 29, 30, 31, 32, 33, 34). We previously uncovered the STRN3-containing PP2A phosphatase accountable for the inactivation of MST1/2 kinases (loss of Hippo signal) in GC and therefore developed the SHAP peptide as a highly selective PP2A inhibitor to restore Hippo signal against GC (16). Moreover, we further revealed that STRIPAK-mediated loss of MST1/2 activity can promote the DNA repair capacity of cancer cells to induce resistance towards radiotherapy and chemotherapy (26). Also, we further developed two STRIPAK assembly inhibitor peptides (SAIP1/2) to disrupt STRIPAK assembly and thereby release MST1/2 to inhibit DNA repair and induce synthetic lethality with PARP inhibitor, that is, sensitize GC towards PARP inhibition (26).

Based on these previous studies, we set out to identify compounds targeting the PP2Aa–STRN3–MST1/2 axis to restore Hippo activity to not only suppress GC growth but also sensitize GC towards chemotherapy. Here, we report FDA-approved drug compound disulfiram (DSF) and inhibitor CX-6258 (CX) able to break the PP2Aa–STRN3–MST1/2 axis, therefore recovering the Hippo signal.

## Results

### AlphaScreen identifies DSF as an inhibitor of the PP2Aa–STRN3 interaction

**Figure 1:**
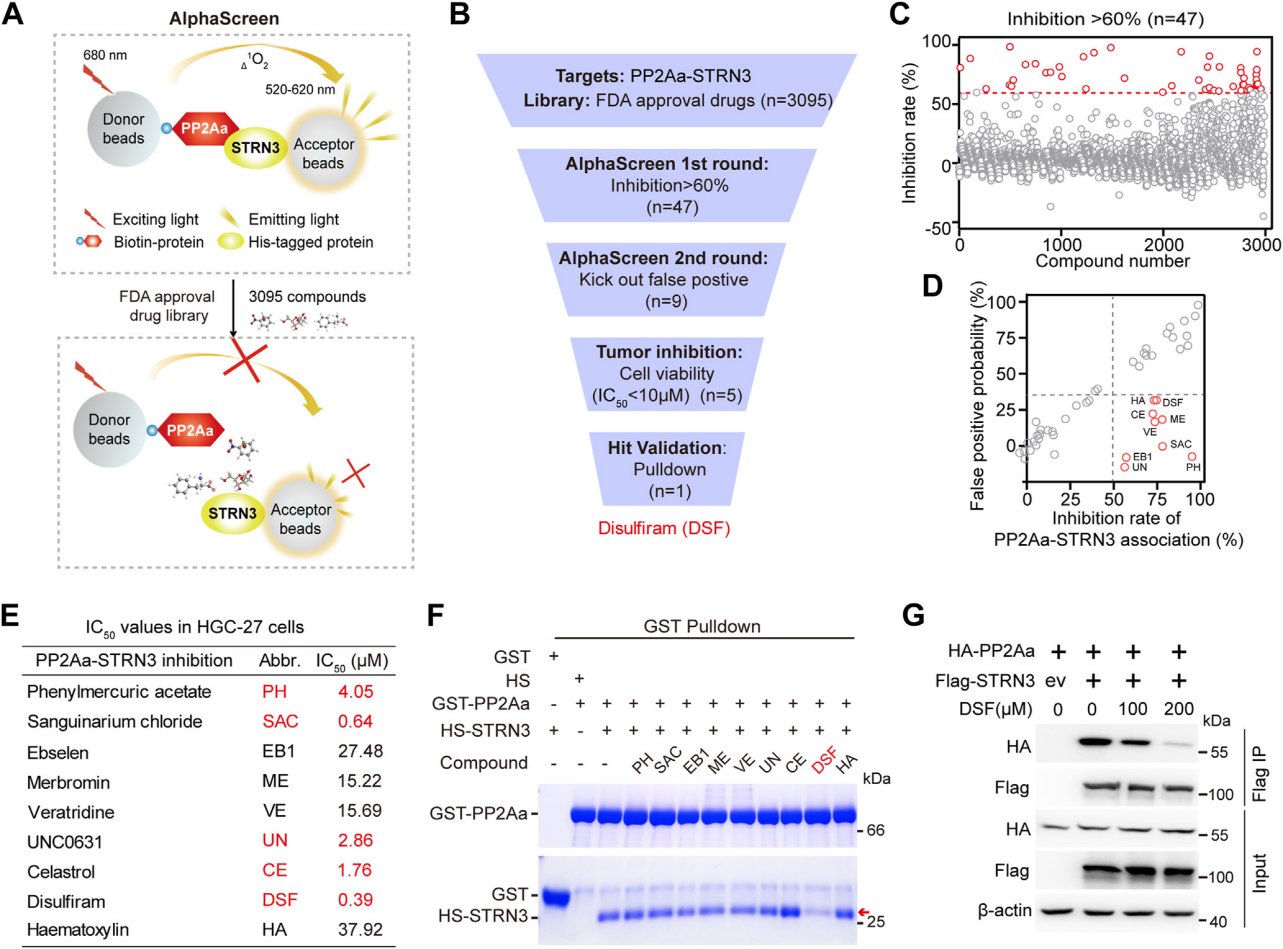
**Identification of DSF as an inhibitor of the PP2Aa–STRN3 interaction.***A*, illustration of the first-round AlphaScreen to find out compounds disrupting PP2Aa–STRN3 interaction. *B*, workflow of four step-by-step compound screening and confirmation strategies. N represents the number of compounds after each screening step. *C*, result of inhibition rate for disrupting PP2Aa–STRN3 interaction in the first round AlphaScreen. *Dash line* refers to inhibition efficiency higher than 60% (n = 47 for *red circle*). *D*, result of the second round AlphaScreen to kick out false positive hits which disrupted interactions between protein and donor/acceptor beads instead of breaking the PP2Aa–STRN3 protein–protein interaction. *Vertical dash line* refers to inhibition efficiency higher than 50% while *horizontal dash line* refers to false positive probability less than 35% (n = 9). *E*, IC_50_ values of nine candidate compounds in HGC-27 cells. The IC_50_ values are less than 5 μM highlighted in *red*. *F*, GST pulldown assay to confirm the disruption effect of PP2Aa–STRN3 association with the indicated 10 μM nine compounds treatment. STRN3 refers to amino acid 64 to 145, hereafter. *G*, co-IP analysis of the interaction between HA-PP2Aa and Flag-STRN3 in HEK293A cells treated with indicated dose of DSF. Co-IP, co-immunoprecipitation; DSF, disulfiram; HS, His-Sumo tag; ev, empty vector.

To identify drug compounds which can mimic the SHAP peptide–induced Hippo reactivation strategy *via* disrupting the PP2Aa–STRN3 interaction (Fig. S1*A*, previous work) (16), we applied a protein–protein interaction-based AlphaScreen platform to screen an FDA-approved drug library containing 3095 chemicals (TargetMol inhibitors) (Fig. 1*A*). Briefly, in the AlphaScreen system, the biotin-labeled recombinant PP2Aa protein was first immobilized on streptavidin donor beads, while purified His-tagged STRN3 protein was bound to the nickel chelate acceptor beads. Upon illumination at 680 nm, the photosensitizer in donor beads converts ambient oxygen to an excited state, singlet molecular oxygen (^1^O_2_). When the distance between PP2Aa–STRN3 is shorter than 200 nm, acceptor beads can receive the ^1^O_2_ resulting in the luminescence signals at 520∼620 nm (35, 36). Thus, compounds able to disrupt the PP2Aa–STRN3 interaction would make the distance between STRN3 and PP2Aa longer than 200 nm, leading to reduced luminescence signal (Fig. 1*A*). The inhibition rate was calculated as the percentage of reduction in luminescence signals to indicate the extent of disruption of the PP2Aa–STRN3 association by compounds (Fig. 1. *B* and *C*).

In the first round AlphaScreen assay (Table S1), we identified 47 compounds able to disrupt the PP2Aa–STRN3 interaction (with an inhibition rate above 60%) from 3095 compounds (Fig. 1. *B* and *C*). To rule out the false positive hits that disrupt interactions between protein and donor/acceptor beads instead of breaking the PP2Aa–STRN3 protein–protein interaction *per se*, we next performed second round screening using both biotin-labeled and His-tagged STRN3 (Fig. S1*B*). By using a criteria of inhibition rate higher than 50% (on the PP2Aa–STRN3 interaction) and false positive probability less than 35% (on the interactions between proteins and beads, Table S2), we thus identified nine candidates (Fig. 1*D*).

Subsequently, we performed a cell viability assay to examine whether each candidate compound could inhibit the growth of a human GC cell line HGC-27. The results showed that five compounds including phenylmercuric acetate, sanguinarium chloride, UNC0631, celastrol, and DSF could significantly reduce the viability of HGC-27 cells (IC_50_ < 5.0 μM) (Figs. 1*E* and S1*C*). In the last step, we performed a GST pulldown assay using recombinant proteins of GST-tagged PP2Aa and His-Sumo-tagged STRN3 to further verify the inhibitory effect of these compounds towards the PP2Aa–STRN3 interaction *in vitro*. This analysis revealed that only DSF, but not the other four chemicals, dramatically reduced the PP2Aa–STRN3 association (Fig. 1*F*), which was further confirmed *via* a dose-dependent suppressive effect (Fig. S1*D*). Next, we performed co-immunoprecipitation (Co-IP) assay to examine whether DSF can disrupt the interaction of PP2Aa with STRN3 *in vivo*. The results showed that DSF treatment dramatically decreased the PP2Aa–STRN3 interaction (Fig. 1*G*). As DSF is a medication with well-established pharmacokinetics that has been used as a treatment for alcohol dependence (37) and a promising cancer-killing drug (38, 39), we focused on DSF as a candidate for subsequent investigation (Fig. S1*E*).

### DSF occupies the STRN3-binding site on PP2Aa to disrupt complex assembly

**Figure 2:**
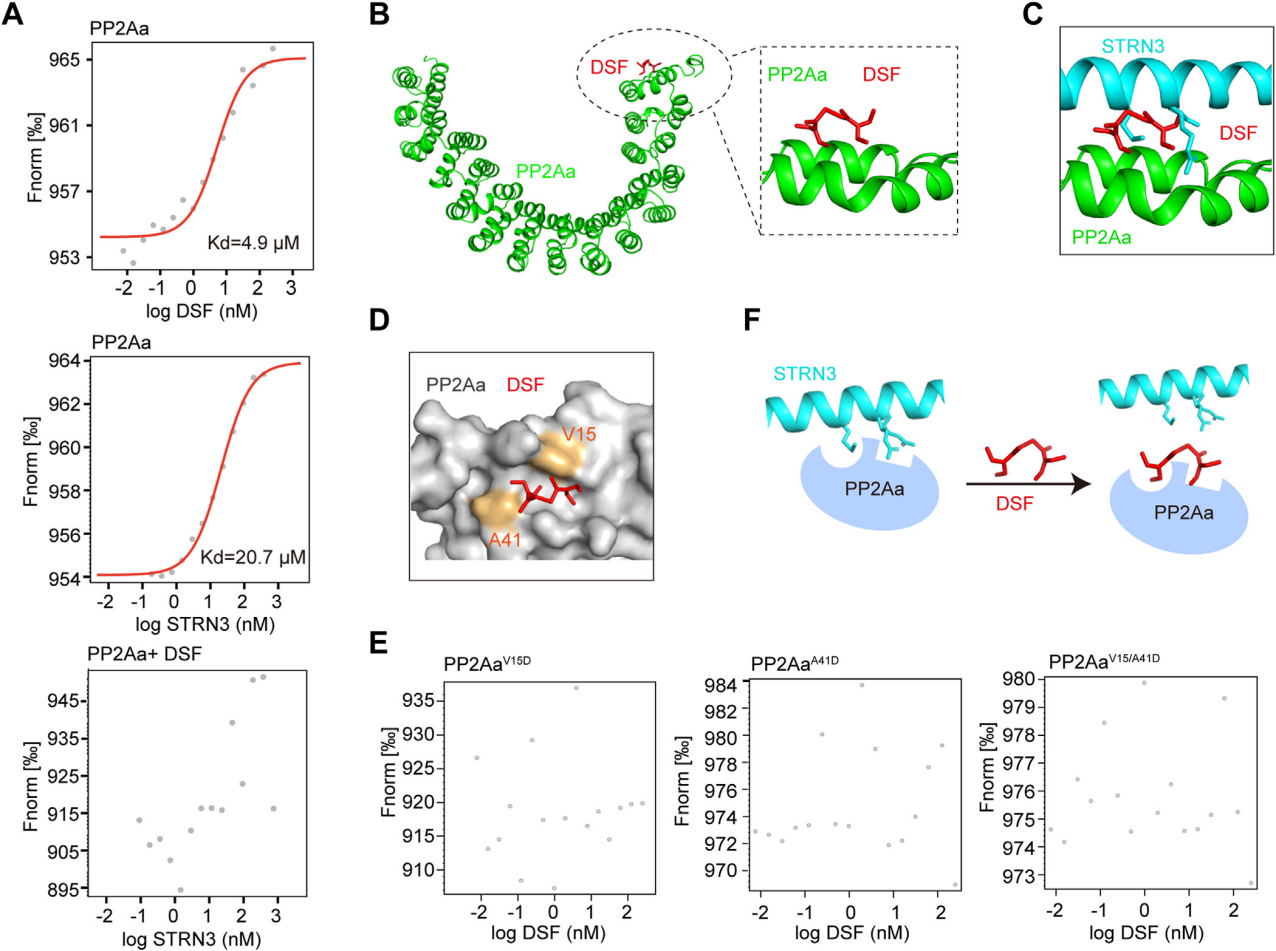
**DSF competes with STRN3 for PP2Aa binding.***A*, detection the DSF–PP2Aa binding affinity and its inhibitory effect on PP2Aa–STRN3 association by MST assay. *B*, virtual docking of DSF binding to first HEAT repeat of the PP2Aa. The PDB ID for crystal structure of the PP2Aa–STRN3 complex is 6IUR. PP2Aa is colored in *green* and shown as cartoon. DSF is shown as *sticks* and colored in *red*. The *right panel* illustrates the binding site of DSF to PP2Aa. *C*, illustration of the occupied position of DSF in PP2Aa–STRN3 complex. *D*, surface structure to highlight the binding pocket (V15, A41) of DSF towards PP2Aa. PP2Aa is shown as surface. *E*, MST assay to show the incapability of PP2Aa mutants in binding to DSF. PP2A^V15/A41D^: PP2Aa mutant variant with V15 and A41 substituted with a glutamate. *F*, model for DSF competing with STRN3 for PP2Aa binding. DSF, disulfiram; MST, microscale thermophoresis.

To define at atomic level the mechanism through which DSF disrupts the PP2Aa–STRN3 interaction, we re-examined the crystal structure of the PP2Aa–STRN3 complex (PDB ID: 6IUR) and found that DSF is likely to bind with PP2Aa but not STRN3 in terms of the molecular shape and electrostatic properties of the complex. To validate this hypothesis, we next performed an isothermal titration calorimetry (ITC) assay to examine whether DSF binds to PP2Aa or STRN3 and found that DSF can specifically bind to PP2Aa but not STRN3 (Fig. S2). More importantly, the binding affinity of DSF to PP2Aa (Kd = 4.1 μM) is about 3-fold higher than that of STRN3 to PP2Aa (Kd = 11.9 μM) (Fig. S2, *A* and *B*). Consistent with these results, a microscale thermophoresis (MST) assay similarly revealed a 4-fold stronger binding affinity of DSF to PP2Aa (Kd = 4.9 μM) than that of STRN3 to PP2Aa (Kd = 20.7 μM) (Fig. 2*A*). Moreover, addition of DSF readily disrupted the binding of STRN3 to PP2Aa, indicating a competitive manner between DSF and STRN3 for PP2Aa binding (Fig. 2*A*).

To further probe the specific binding mode of DSF with PP2Aa, we utilized virtual molecular docking to explore the details of DSF–PP2Aa-binding interface (40) and thus observed that DSF may embed into a groove on the molecular surface of PP2Aa (Fig. 2*B*). Notably, this groove of PP2Aa was found just next to the interface of the PP2Aa–STRN3 complex, further supporting the notion that DSF may compete with STRN3 for binding PP2Aa. Further structural comparison of PP2Aa in complex with STRN3 or DSF revealed that DSF binds to PP2Aa *via* similar position and manner as does the key residues of STRN3 (Fig. 2*C*). According to our previous mutational studies of the PP2Aa–STRN3 complex (16) as well as the current docking results, we reasoned that the amino acid residues V15 and A41 delineating the STRN3-binding pocket of PP2Aa may be also required for binding DSF (Fig. 2*D*). To verify this possibility, we generated two PP2Aa mutant variants with either V15 or A41 substituted with a glutamate (hereafter referred to as V15D and A41D). By introducing electrostatic perturbation into the hydrophobic pocket, we speculated that these PP2Aa mutant variants could no longer bind with DSF. Indeed, MST assay showed that mutation of either V15D, A41D, or in combination completely abolished the ability of PP2Aa to bind DSF (Fig. 2*E*). Taken together, these results demonstrated that DSF occupies the STRN3-binding pocket on PP2Aa, thus preventing STRN3 from binding to PP2Aa (Fig. 2*F*).

### DSF reactivates Hippo to suppress GC growth

**Figure 3:**
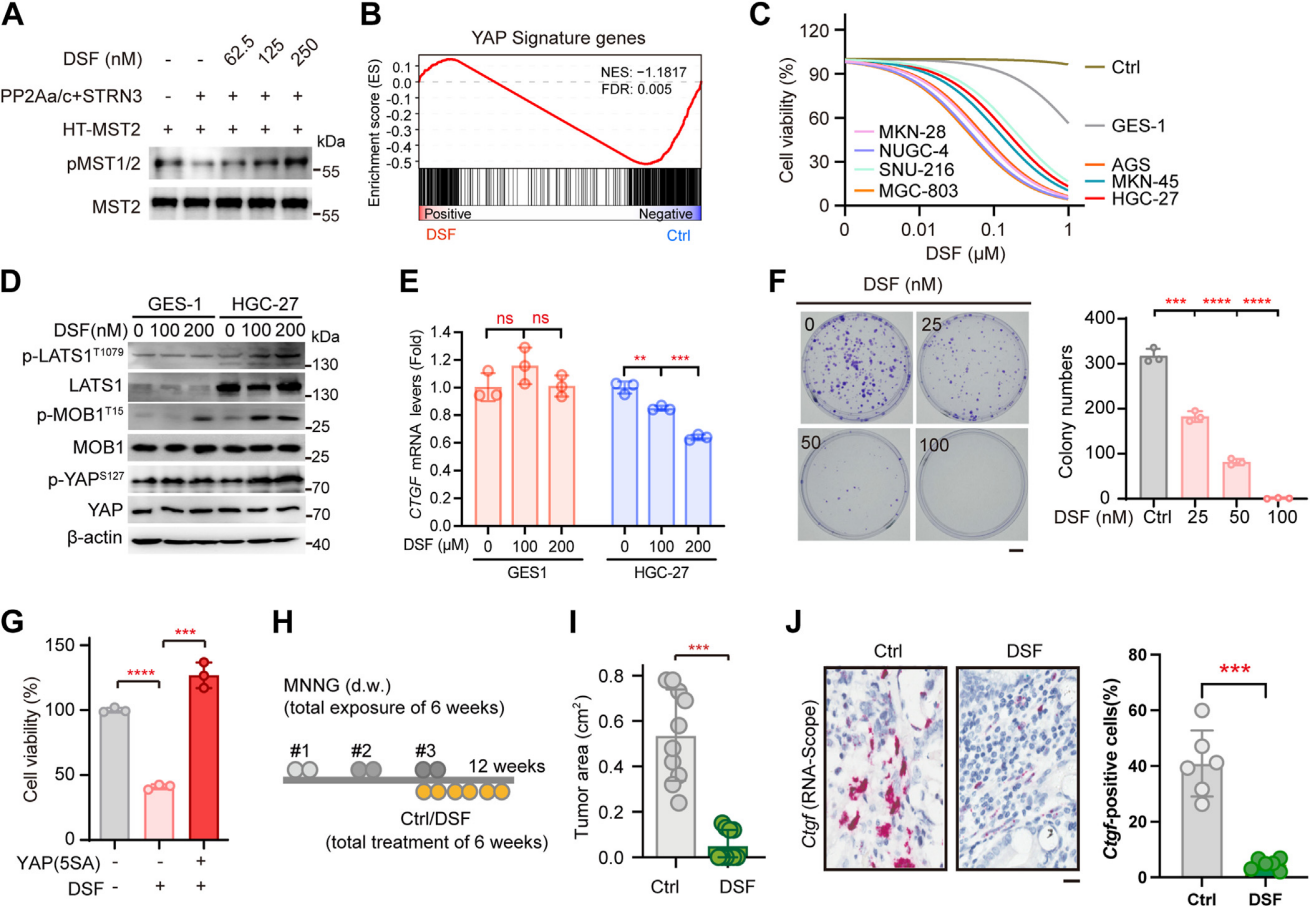
**DSF acts as a Hippo activator to inhibit GC.***A*, *in vitro* dephosphorylation assay to confirm the dose-dependent effects of DSF on the phosphatase activity of PP2Aa/c core enzyme using pMST2 (T180) as a substrate. *B*, gene set enrichment analysis (GSEA) of YAP signature genes in DSF-treated HGC-27 cells for 48 h. Normalized enrichment score (NES) and FDR values are indicated. *C*, cell viability of seven different GC cell lines (MKN-28, NUGC-4, SNU-216, MGC-803, AGS, MKN-45, and HGC-27) and one non-cancerous gastric epithelial cell line GES-1 towards DSF treatment. *D*, immunoblots of the phosphorylation levels of Hippo components including LATS1, MOB1, and YAP in GES-1 and HGC-27 cells with different doses of DSF treatment. *E*, mRNA levels of *CTGF* in GES1 and HGC-27 cells treated with indicated dose of DSF for 48 h (n = 3). Data are presented as means ± SD. The data were analyzed using one-way ANOVA, followed by the Tukey’s post hoc test. ∗∗*p* < 0.01; ∗∗∗*p* < 0.001; n.s., no significance. *F*, the colony formation of HGC-27 cells treated with DSF of different doses (n = 3). The *right bar* figure represents the statistics of colony numbers. Data are presented as means ± SD. The data were analyzed using one-way ANOVA, followed by the Tukey’s post hoc test. ∗∗∗*p* < 0.001; ∗∗∗∗*p* < 0.0001. *G*, cell viability of HGC-27 cells treated with or without DSF after transfected with empty vector and YAP (5SA) plasmid for 48 h (n = 3). YAP (5SA): a constitutively active form of YAP (S61A/S109A/S127A/S164A/S381A). Data are presented as means ± SD. The data were analyzed using one-way ANOVA, followed by the Tukey’s post hoc test. ∗∗∗*p* < 0.001; ∗∗∗∗*p* < 0.0001. *H*, schematic illustration of treating MNNG-induced GC mice with DSF. #1, #2, and #3 indicate first, second, and third round of MNNG treatment, respectively. *Gray circle* above the line represents 1-week duration of MNNG treatment; *yellow circle* below the line represents 1-week duration of DSF treatment. *I*, areas of MNNG-induced GC tumors in control mice and those treated with DSF (n = 10 per group). Data are presented as means ± SD. Significance was tested using unpaired *t* test, ∗∗∗*p* < 0.001. *J*, RNA-Scope-IHC analysis and quantification of *Ctgf*-positive cells of MNNG-induced GC tumors in control mice and those treated with DSF. Scale bar represents 10 μm. Data are presented as means ± SD. Significance was tested using unpaired *t* test, ∗∗∗*p* < 0.001. DSF, disulfiram; GC, gastric cancer; MNNG, 1-Methyl-3-nitro-1-nitrosoguanidine.

Given the above findings that DSF can disrupt the assembly of PP2Aa–STRN3 complex, we expected the compound would thus relieve the inhibitory effect of PP2A phosphatase towards MST1/2 kinases, thus suppressing oncogenic YAP signaling and cancer cell proliferation. To this end, we first performed *in vitro* dephosphorylation assay to examine the activation of MST1/2 kinases (*i.e.*, the phosphorylation level of MST1/2, hereafter referred as pMST1/2). Briefly, purified recombinant protein of MST2 was first auto-phosphorylated and then subjected to dephosphorylation by the reconstituted PP2Aa–STRN3–PP2Ac holophosphatase *in vitro*, with the residual phosphorylation level of MST1/2 being monitored to reflect the phosphatase activity. As expected, we found that the pMST1/2 was reduced in the presence of the PP2Aa–STRN3–PP2Ac holophosphatase (Fig. 3*A*, lane 1 *versus* lane 2). Importantly, addition of DSF into the system dramatically recovered the pMST1/2 following a dose-dependent manner (Fig. 3*A*).

Next, we performed an RNA-seq whole-transcriptome analysis of HGC-27 cells, in which Hippo signal was found to be diminished due to elevated STRN3 expression. In cells treated with DSF, a total of 220 genes were significantly altered, among which 60 genes were upregulated and 160 genes were downregulated (Fig. S3*A*). Kyoto encyclopedia of genes and genome (KEGG) analysis and gene set enrichment analysis (GSEA) revealed a significantly negative correlation of DSF treatment with Hippo target gene expression (Figs. 3*B* and S3*B*). In addition, we also observed significant changes for expression of genes involved in DNA replication and platinum drug resistance, implying a role for DSF in DNA repair–related chemoresistance (Fig. S3*B*).

DSF has been reported to inhibit the Src kinase activity (41), which otherwise can also activate YAP activity *via* phosphorylation of Y341/Y357/Y397 residues (42). To pinpoint whether the inhibitory effect of DSF on YAP inactivation is dependent on the STRIPAK-Hippo signaling or on the Src–YAP axis in GC, we examined DSF-induced YAP phosphorylation by using antibodies which specifically recognize phosphorylated serine (pSer) or phosphorylated tyrosine (pTyr). Interestingly, this analysis revealed that DSF dose-dependently increased the level of pYAP^Ser^ but only had a marginal effect on pYAP^Tyr^ (Fig. S3*C*).

Considering the well-characterized tumor suppressor function of the MST1/2 kinases, it is conceivable that DSF may eventually inhibit GC growth through reactivation of MST1/2. To examine the cytotoxic effect of DSF, we tested seven different GC cells lines (MKN-28, NUGC-4, SNU-216, MGC-803, AGS, MKN-45, and HGC-27) and one non-cancerous gastric epithelial cell line GES-1. The results showed that DSF markedly inhibited the viability of the seven GC cell lines tested, with IC_50_ values ranging from 40 nΜ to 190 nΜ (Figs. 3*C* and S3*D*). In contrast, GES-1 cells were relatively resistant to DSF treatment showing an IC_50_ value of 1.29 μΜ ((Figs. 3*C* and S3*D*), results suggesting a selective cytotoxic effect of DSF toward GC and a therapeutic window toward GC treatment. To validate this, we examined the Hippo signaling in DSF-treated GES-1 and HGC-27 cells and found that DSF dose-dependently promoted the phosphorylation of LATS1, MOB1, and YAP in HGC27 cells but not GES-1 cells, results indicative of selectively reactivating Hippo signaling in cancer (Fig. 3*D*). Consistent with these observations, DSF treatment also significantly reduced the mRNA levels of YAP target genes *CTGF* in HGC-27 but not GES1 cells (Fig. 3*E*).

In addition, we performed colony formation assay to verify the long-term effect of DSF on GC growth and consistently observed that DSF significantly inhibited colony formation of HGC-27 cells in a dose-dependent manner (Fig. 3*F*). Moreover, transfection of a constitutively active form of YAP (5A mutant) fully rescued DSF-inhibited growth of GC cells, again confirming the restoration of the suppressive effect of Hippo signal on YAP activity upon DSF treatment (Fig. 3*G*). Given that DSF can act as copper ionophore (43), we also assessed the potential effect of copper ion on the antitumor capacity of DSF. The results showed that addition of copper ion further dramatically enhanced such suppressive effect of DSF on HGC-27 cell viability (Fig. S3, *E* and *F*).

To further evaluate the antitumor efficacy of DSF *in vivo*, we applied a chemical carcinogen (1-Methyl-3-nitro-1-nitrosoguanidine, MNNG)-induced GC model following a standard procedure (44). Briefly, drinking water containing MNNG was served to mice for 2 weeks and then normal water for the next 2 weeks. After a 6-week period of MNNG treatment, the mice were further treated with or without DSF intravenously once tumors became palpable (Fig. 3*H*). Consistently, the area of tumors in mice receiving DSF was found to be substantially smaller than those in control mice (Fig. 3*I*). These observations were further confirmed by sharp decrease of *Ctgf* expression shown by RNA-Scope analysis (Fig. 3*J*). Taken together, these results demonstrated a therapeutic effect of DSF on selective inhibition of GC but not normal cell growth through reactivating Hippo kinases.

### DSF inhibits DNA repair and sensitizes GC to chemotherapy

**Figure 4:**
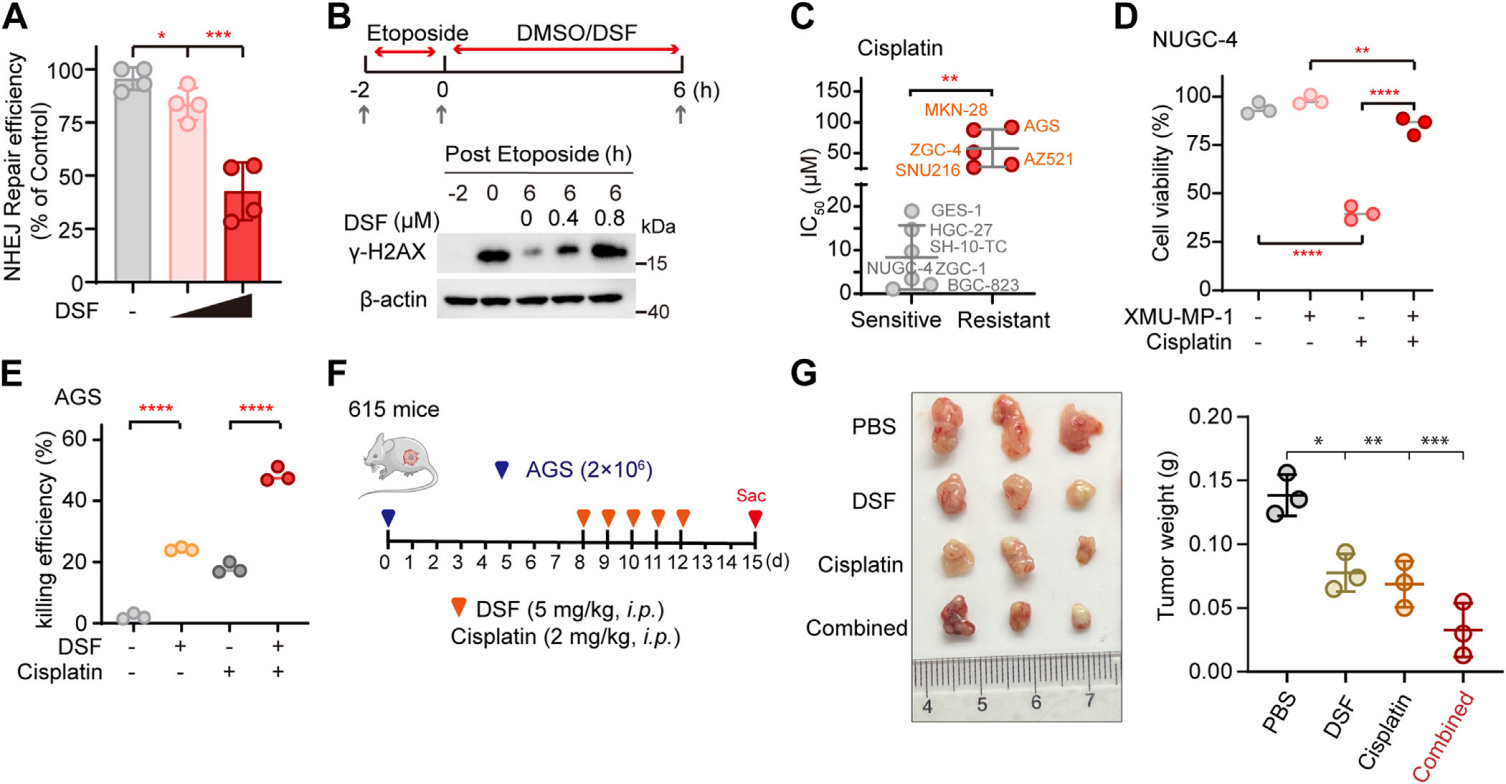
**DSF sensitizes GC to cisplatin *via* limiting DNA repair capacity.***A*, HGC-27 cells treated with increased dose of DSF were subjected to NHEJ reporter assay (n = 4). Data are presented as means ± SD. Significance was tested using one-way ANOVA, followed by the Tukey’s post hoc test. ∗*p* < 0.05; ∗∗∗*p* < 0.001. *B*, immunoblots of γ-H2AX expression incubated with or without DSF. Experimental workflow of DSF treatment in HEK293A cells pre-treated with 20 μM etoposide for 2 h. *C*, IC_50_ values in the indicated different cell lines treated with cisplatin. The cells were divided into two groups: cisplatin sensitive or resistant. Data are presented as means ± SD. Significance was tested using unpaired *t* test, ∗∗*p* < 0.01. *D*, cell viability of NUGC-4 treated with XMU-MP-1 and cisplatin or combination (n = 3). Significance was tested using one-way ANOVA, followed by the Tukey’s post hoc test. ∗∗*p* < 0.01; ∗∗∗∗*p* < 0.0001. *E*, killing efficiencies of AGS cells treated with 0.05 μM DSF and 20 μM cisplatin or in combination (n = 3). Significance was tested using one-way ANOVA, followed by the Tukey’s post hoc test. ∗∗∗∗*p* < 0.0001. *F*, schematic illustration of the tumor formation assay *in vivo*. *G*, photograph showing tumors in mice bearing AGS cells were intratumorally administered DSF and cisplatin, either alone or in combination (*left panel*). Tumor weight was measured in the indicated groups (*right panel*). Significance was tested using one-way ANOVA, followed by the Tukey’s post hoc test. ∗*p* < 0.05; ∗∗*p* < 0.01; ∗∗∗*p* < 0.001. DSF, disulfiram; GC, gastric cancer; NHEJ, nonhomologous end-joining.

In addition to suppress cell growth, we previously also revealed that the MST1/2 kinases, once released from STRIPAK complex, could enter the nucleus to suppress DNA repair upon chemotherapy treatment, and loss of the Hippo signal resulted in acquired drug resistance (26). Based on the RNA-seq analysis in DSF-treated HGC-27 cells (Fig. S3*B*), we speculated that DSF-mediated MST1/2 activation may also enhance the sensitivity of GC cells towards chemotherapy *via* inhibiting DNA repair ability. To test this hypothesis, we first examined the DNA repair efficiency in DSF-treated cells using a well-established DNA double strand break reporter system (45, 46). Indeed, treatment with DSF greatly suppressed nonhomologous end-joining repair capacity in a dose-dependent manner (Fig. 4*A*). Moreover, we also treated HEK293A cells with etoposide for 2 h to induce DNA damage as shown by increased levels of γ-H2AX, a DNA damage marker; and the cells were further incubated with or without DSF for another 6 h (Fig. 4*B*, upper panel). Consistent with our notion that DSF inhibits DNA repair, we found the damaged DNA was repaired much more slowly (as measured by residual γ-H2AX level) in DSF-treated cells when compared with the control DMSO-treated cells (Fig. 4*B*).

Given our findings that DSF can reactivate MST1/2 kinases and inhibit DNA repair, we next assess the possible correlation of MST1/2 kinases activities with chemotherapy resistance. To this end, we first examined the IC_50_ values of 11 different GC cells towards cisplatin, a first-line chemotherapy drug. Based on the IC_50_ values, we divided the 11 GC cell lines into two groups: cisplatin-sensitive (IC_50_ < 20 μΜ) and cisplatin-resistant (IC_50_> 20 μΜ). We found the cell lines with relatively higher levels of pMST1/2 such as NUGC-4 were more sensitive to cisplatin, while the cell lines with lower levels of pMST1/2 such as AGS, AZ521, and MKN-28 were more resistant to cisplatin (Figs. 4*C* and S4*A*). Moreover, co-treatment of NUGC-4 cells with XMU-MP-1, a MST1/2 kinase inhibitor (47), triggered much more resistance to cisplatin (Fig. 4*D*).

Considering AGS and MKN-28 cells are intrinsically resistant to cisplatin and that DSF could restore the MST1/2 kinases activities, we thus used these two cell lines to evaluate whether targeting to recover pMST1/2 by DSF could enhance cisplatin sensitivity. Notably, cisplatin alone or DSF alone exhibited only about 20% efficiency in killing cancer cells with nontoxic concentrations, yet combining DSF with cisplatin resulted in about 50% death of AGS or MKN-28 cells (Figs. 4*E* and S4*B*). To further investigate the combined therapeutic effect of cisplatin and DSF *in vivo*, we next compared the tumor formation abilities using a xenograft mouse model. Briefly, we first injected mice subcutaneously with AGS cells for 1 week to allow tumor growth until they were palpable, then the mice were intratumorally injected with either cisplatin or DSF or a combination of both drugs, for another five consecutive days and harvested the tumors at 15 days post tumor transplantation (Fig. 4*F*). While cisplatin or DSF treatment yielded modestly lower tumor weights than that of control treatment, combination of cisplatin and DSF almost diminished tumor growth (Fig. 4*G*), illustrating elevated Hippo kinase activity *via* DSF re-sensitized tumors to cisplatin resistance.

### The DSF structural analog thiram functions similarly as a Hippo activator

**Figure 5:**
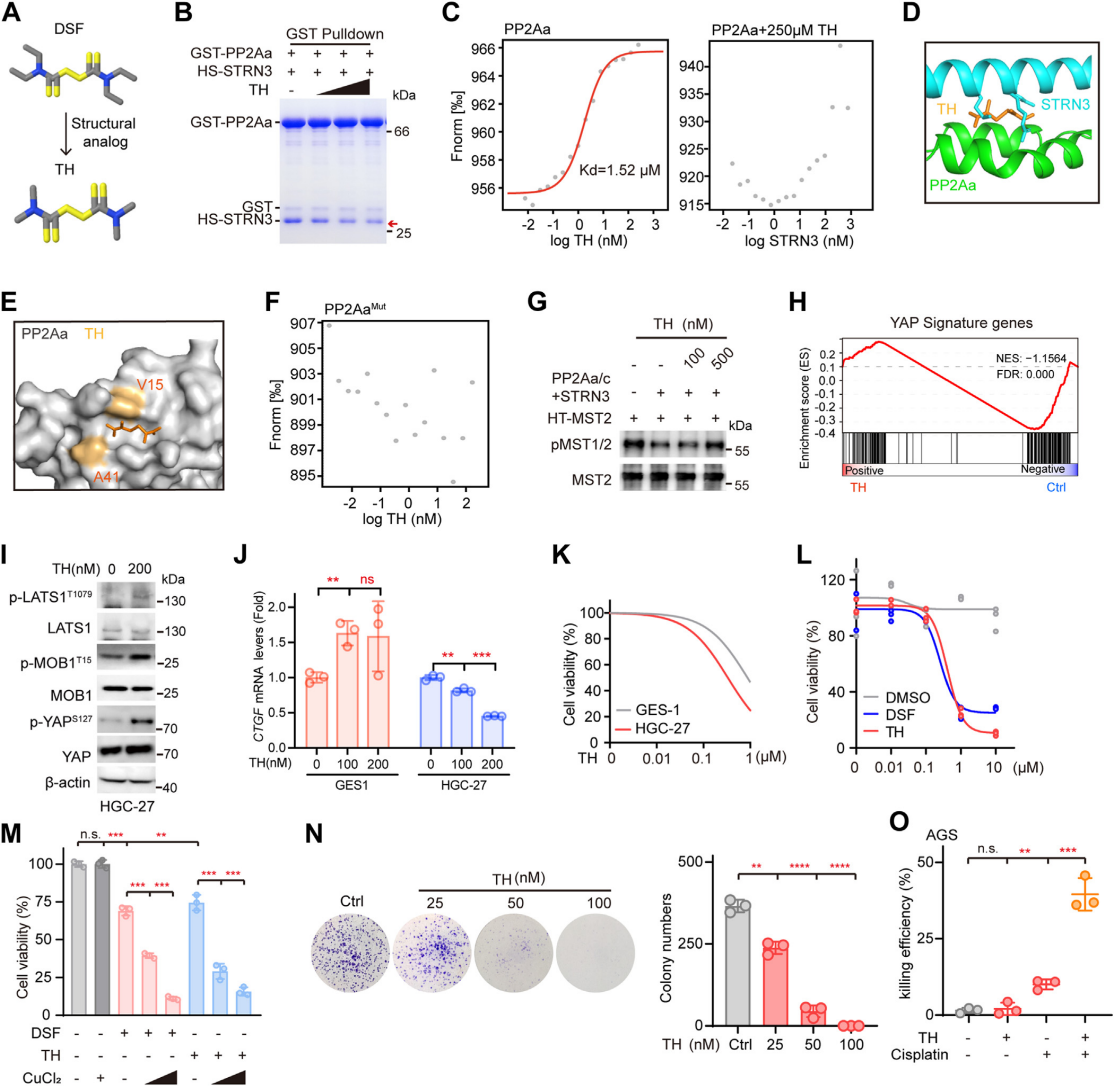
**TH acts as a structural analog of DSF to inhibit GC.***A*, the chemical structures of DSF and TH. *B*, GST pulldown assay to demonstrate the dose-dependent disruption effect of TH on PP2Aa–STRN3 association. The dosage for TH is 0, 2.5, 5, 10 μM, respectively. *C*, MST assay to show the interruption effect and association between TH and PP2Aa. *D*, virtual docking of TH binding to first HEAT repeat of the PP2Aa-occupied STRN3-binding site. STRN3 was colored by *cyan* while PP2Aa was colored by *green*. *E*, surface structure to highlight the binding pocket (V15, A41) of TH towards PP2Aa. PP2Aa was shown as surface. *F*, detection of the disruption effect of PP2Aa mutant in binding with DSF by MST assay. PP2A^Mut^: PP2Aa mutant variant with V15 and A41 substituted with a glutamate. *G*, *in vitro* dephosphorylation assay to confirm dose-dependently effects of TH on the phosphatase activity of PP2Aa/c core enzyme using pMST2 (T180) as a substrate. *H*, GSEA analysis of YAP signature genes in TH-treated HGC-27 cells for 48 h. Normalized enrichment score (NES) and FDR values are indicated. *I*, immunoblots of the phosphorylation level of Hippo components in HGC-27 cells treated with or without TH (200 nM). *J*, relative mRNA levels of *CTGF* in GES-1 and HGC-27 cells treated with TH for 48 h (n = 3). Significance was tested using one-way ANOVA, followed by the Tukey’s post hoc test. ∗∗*p* < 0.01; ∗∗∗*p* < 0.001. *K*, cell viability of two different cell lines (HGC-27 and GES-1) with TH treatment (n = 3). *L*, cell viability of HGC-27 cells treated with DSF or TH (n = 3). *M*, cell viability of HGC-27 cells treated with DSF/TH or DSF/TH-Cu complex (n = 3). Data are presented as means ± SD. The data were analyzed using one-way ANOVA, followed by the Tukey’s post hoc test. ∗∗*p* < 0.01; ∗∗∗*p* < 0.001. *N*, the colony formation of HGC-27 cells treated with TH of different doses (n = 3). The *right bar* figure represents the statistics of colony numbers. Data are presented as means ± SD. The data were analyzed using one-way ANOVA, followed by the Tukey’s post hoc test. ∗∗*p* < 0.01; ∗∗∗∗*p* < 0.0001. *O*, killing efficiencies of AGS cells treated with 0.1 μM TH and 10 μM cisplatin alone or in combination. Significance was tested using one-way ANOVA, followed by the Tukey’s post hoc test. ∗∗*p* < 0.01; ∗∗∗*p* < 0.001; n.s., no significance. DSF, disulfiram; GC, gastric cancer; GSEA, gene set enrichment analysis; MST, microscale thermophoresis.

Encouraged by the antitumor effect of DSF and its combination with cisplatin, we went on to screen structural analogs of DSF and speculated that they could exert similar functions as MST1/2 kinases agonist. To this end, we used the structural formula of DSF as a model to query the compounds showing 90% similarity to DSF and thus obtained 104 compounds. Next, we selected the top six compounds for further analysis (Fig. S5*A*). Similar to DSF, we found all these six compounds could dose-dependently inhibit HGC-27 cancer cell growth, with CAS 5675-76-3 having the strongest killing effect (Fig. S5*B*). Interestingly, we observed that thiram (TH) shows the highest similarity with DSF and was thus chosen as an example to illustrate whether these analogs had similar functions as DSF (Fig. 5*A*).

First, we examined whether TH can also disrupt the PP2Aa–STRN3 interaction. Indeed, both GST pulldown and MST assays showed that TH can efficiently disrupt the interaction between STRN3 and PP2Aa in a dose-dependent manner (Fig. 5, *B* and *C*), which was further validated by Co-IP assay performed in HEK293A cells (Fig. S5*C*). Moreover, TH can also directly bind to PP2Aa with a Kd value of 1.52 μM (Fig. 5*C*), a 14-fold stronger binding affinity than that between STRN3 and PP2Aa (Kd = 20.7 μM, Fig. 2*A*). Next, we performed virtual molecular docking of TH onto PP2Aa (Fig. S5*D*) and found that TH also tends to embed into the PP2Aa pocket which was formed by amino acid residues V15 and A41 and was shown to accommodate STRN3 or DSF (Fig. 5, *D* and *E*). As expected, MST assay showed that mutation of V15D, A41D, or in combination completely abolished the ability of PP2Aa to bind TH (Figs. 5*F* and S5*E*). Therefore, we concluded that TH occupied the STRN3-binding pocket on PP2Aa to prevent STRN3 binding, as did DSF (Fig. 5*D*). In addition, the *in vitro* dephosphorylation assay reproducibly demonstrated that TH could dose-dependently recover the phosphorylation levels of MST1/2 kinases (Fig. 5*G*).

Subsequently, we also performed an RNA-seq whole-transcriptome analysis in HGC-27 cells treated with TH, which showed a total of 90 genes were significantly altered, with 44 and 46 genes upregulated and downregulated, respectively (Fig. S5*F*). Further GSEA also revealed a negative correlation of TH treatment with YAP signature genes’ expression (Fig. 5*H*). To further verify the regulatory effect of TH on Hippo pathway, we treated HGC-27 cells with TH and found an increase in the phosphorylation levels of LATS1, MOB1, and YAP (Fig. 5*I*), as well as the mRNA expression levels of *CTGF*, in a dose dependent manner (Fig. 5*J*). In sharp contrast, no significant change was detected for mRNA expression levels of *CTGF* in GES-1 cells treated with TH (Fig. 5*J*).

Also, we examined the inhibitory effect of TH on GC cell growth and found that TH markedly reduced the viability of HGC-27 cells with an IC_50_ of 0.4 μM, which is comparable to that of DSF (Fig. 5, *K* and *L*). Given that DSF inhibition of GC can be greatly enhanced by copper ion as well as that TH can also form stable complex with copper ion (48), we then assessed the potential effect of copper ion on the antitumor capacity of TH (49) and similarly revealed addition of copper ion markedly enhanced TH inhibition of GC cell growth (Figs. 5*M* and S5*G*). At last, we performed colony formation assay to verify the long-term effect of TH treatment on GC cell growth and found that TH significantly inhibited colony formation of HGC-27 cells (Fig. 5*N*). In addition, we also compared the repair dynamic of HGC-27 cells pre-treated with etoposide for 2 h before incubated with or without TH for another 6 h to evaluate its inhibitory effect on DNA repair. In extremely resemble to DSF, we found the DNA repair capacity of GC cells was decreased upon TH treatment (Fig. S5*H*). Consistent with this observation, TH treatment also significantly sensitized GC cells to cisplatin (Fig. 5*O*).

Taken together, these results demonstrated that TH, as a structural analog of DSF, can not only reactivate MST1/2 kinases *via* disrupting the PP2Aa–STRN3 interaction to limit GC growth but also sensitize GC to chemotherapy *via* inhibiting DNA repair, results again confirming the feasibility and efficacy of targeting the PP2Aa–STRN3 interaction in the STRIPAK complex.

### AlphaScreen identifies CX as an inhibitor of the STRN3–MST1/2 interaction

**Figure 6:**
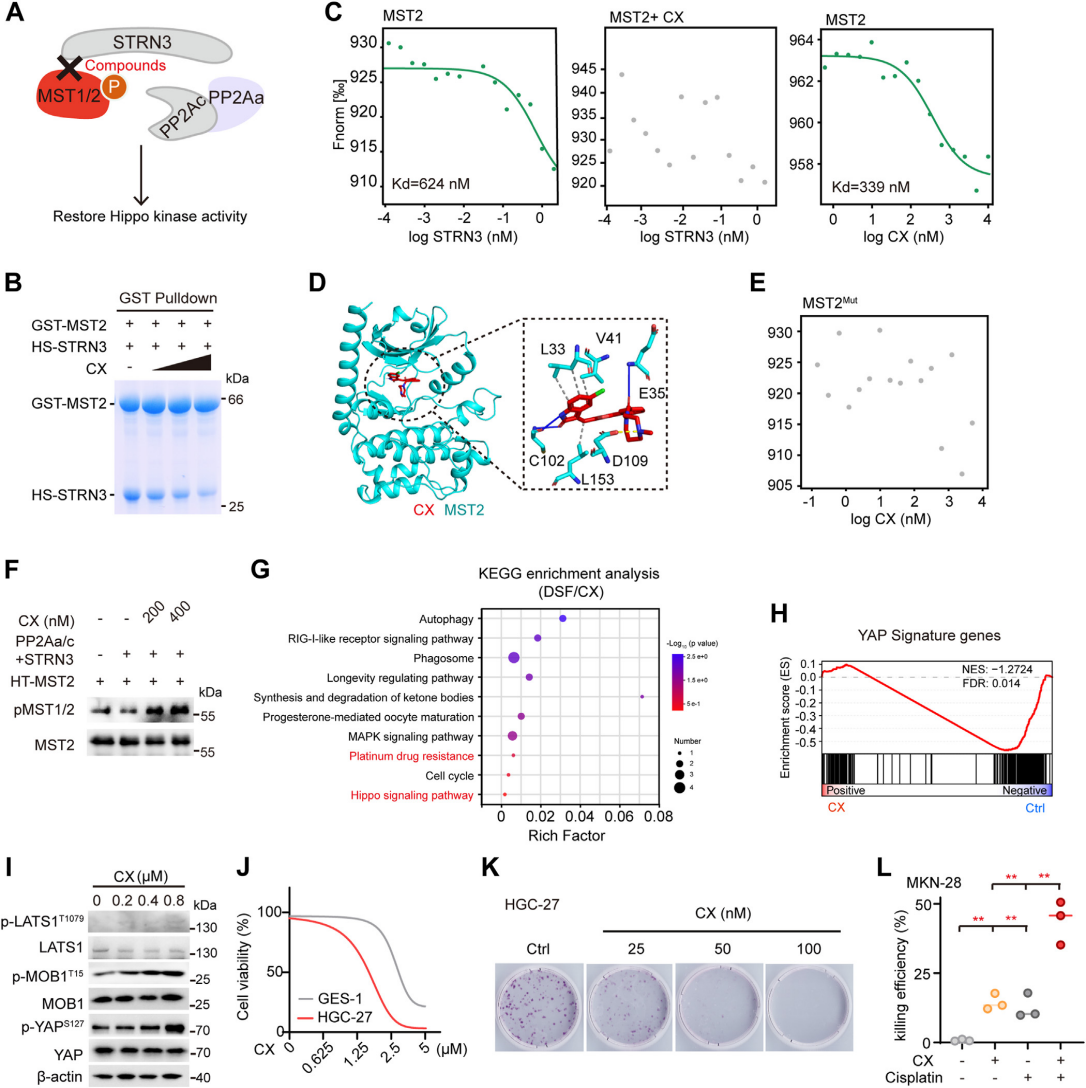
**CX disrupts the STRN3–MST2 interaction to recover YAP activity.***A*, graphic illustration for compounds used to disrupt STRN3–MST2 interaction for restoration of MST1/2 kinases. *B*, GST pulldown assay to confirm the disruption effect of CX on STRN3-MST2 in different doses (0, 2.5, 5, 10 μM). *C*, measurement of CX-MST2 binding affinity and its inhibitory effect on STRN3-MST2 association by MST assay. *D*, virtual docking of CX binding to the ATP-binding pocket of MST2. *Gray dash line*: hydrophobic interaction; *blue line*: hydrogen bond; *yellow dash line*: salt bridge. *E*, MST assay to detect the binding efficiency of CX towards the MST2 mutant. MST2^Mut^ refers to L33/E35/V41A mutant of MST2. *F*, *in vitro* dephosphorylation assay to confirm dose-dependently effects of CX on the phosphatase activity of PP2Aa/c core enzyme using pMST2 (T180) as a substrate. *G*, KEGG enrichment analysis of altered pathways enriched of common 60 altered genes in DSF/CX-treated HGC-27 cells. *H*, GSEA analysis of YAP signature genes in CX-treated HGC-27 cells for 48 h. *I*, immunoblots of the phosphorylation level of Hippo components in HGC-27 cells treated indicated dose of CX. *J*, cell viability of two different cell lines (HGC-27 and GES-1) with CX treatment (n = 3). *K*, the colony formation of HGC-27 cells treated with DSF of different doses. *L*, killing efficiencies of MKN-28 cells treated with 1 μM CX and 20 μM cisplatin or in combination. Significance was tested using one-way ANOVA, followed by the Tukey’s post hoc test. ∗∗*p* < 0.01. CX, CX-6258; HS, His-Sumo-tagged; DSF, disulfiram; GSEA, gene set enrichment analysis; KEGG, Kyoto encyclopedia of genes and genome; MST, microscale thermophoresis.

After the verification of the PP2Aa–STRN3–based targeting strategy, we reasoned that the compounds disrupting another sub-complex within STRIPAK, that is, STRN3–MST1/2 interaction, would have similar effect with or even better selectivity (Fig. 6*A*). To this end, we subjected the AlphaScreen assay using purified STRN3 and MST2 recombinant proteins against the same library to identify compounds which are able to disrupt the STRN3–MST2 interaction (Fig. 1*A*). Following a similar two-round AlphaScreen selection (Tables S3 and S4), we identified ten candidate compounds which might disrupt the STRN3–MST2 interaction (Fig. S6, *A* and *B*). A subsequent MBP pull-down assay validated three compounds including CX-6258 (CX), suramin sodium salt, and azeliragon, which were able to disrupt the STRN3–MST2 interaction (Fig. S6*C*). Further cell viability assay showed that CX, but not suramin sodium salt or azeliragon, can significantly inhibit HGC-27 cell growth (Fig. S6, *D* and *E*).

Next, GST pull-down and Co-IP assays further confirmed that CX could disrupt the MST2–STRN3 interaction in a dose-dependent manner (Figs. 6*B* and S6*F*). Consistently, MST assay showed that STRN3 and MST2 could not bind with each other in the presence of CX (Fig. 6*C*). Moreover, CX can directly bind to MST2 with a Kd value of 339 nM, a relatively higher binding affinity than STRN3 (Kd = 624 nM) and that CX may compete with STRN3 for binding MST2 (Fig. 6*C*). Further molecular docking suggested that CX may contact the side chain of amino acid residues L33, V41, and L153 of MST2 through hydrophobic interactions on one hand and form hydrogen bond or salt bridge with amino acid residues E35, C102, and D109 of MST2 on the other (Fig. 6*D*). To verify the docking result, we constructed a mutant variant of MST2 with L33/E35/V41 substituted with alanine (referred as MST2^Mut^) and showed that the MST2^Mut^ failed to bind with CX analyzed by MST assay (Fig. 6*E*).

### CX reactivates Hippo to inhibit GC growth and sensitize GC to cisplatin

Consistent to its blocking effect on the STRN3–MST2 interaction, our *in vitro* dephosphorylation assay showed that CX recovered the phosphorylation level of MST2 in a dose-dependent manner (Fig. 6*F*). A subsequent RNA-seq analysis in CX-treated HGC-27 cells identified a total of 374 significantly altered genes, among which 47 were upregulated and 327 were downregulated (Fig. S6*G*). We then compared the RNA-seq results obtained in DSF- and CX-treated cells and identified 60 genes with expression significantly altered in both cases (Fig. S6*H*). Further Kyoto encyclopedia of genes and genome analysis of these 60 genes enriched the Hippo signaling pathway and platinum drug resistance (Fig. 6*G*). GSEA analysis revealed a negative correlation of CX treatment with YAP target genes’ expression (Fig. 6*H*).

Supporting its role of reactivating Hippo, CX treatment enhanced the phosphorylation levels of LATS1, MOB1, and YAP in a dose-dependent manner in HGC-27 cells (Fig. 6*I*). Meanwhile, CX treatment also significantly dose-dependently decreased the mRNA levels of YAP target genes *CTGF* in HGC-27 cells (Fig. S6*I*). Moreover, CX treatment inhibited the cell viability (Fig. 6*J*) and colony formation (Figs. 6*K* and S6*J*) of HGC-27 cells. In addition, CX displayed an inhibition rate towards HGC-27 cells comparable with DSF (Fig. S6*K*). To further assess the potential regulatory effects of CX on DNA repair and drug resistance, we examined γ-H2AX levels in CX-treated 293A cells and found that CX markedly attenuated the repair of cisplatin-induced DNA damage (Fig. S6*L*). Consistently, CX greatly sensitized GC cells to cisplatin as shown by dramatically increased killing efficiency of cisplatin in combination with CX (Fig. 6*L*). Taken together, these results validated the feasibility and efficacy of targeting the STRN3–MST1/2 interaction in the STRIPAK complex.

## Discussion

**Figure 7:**
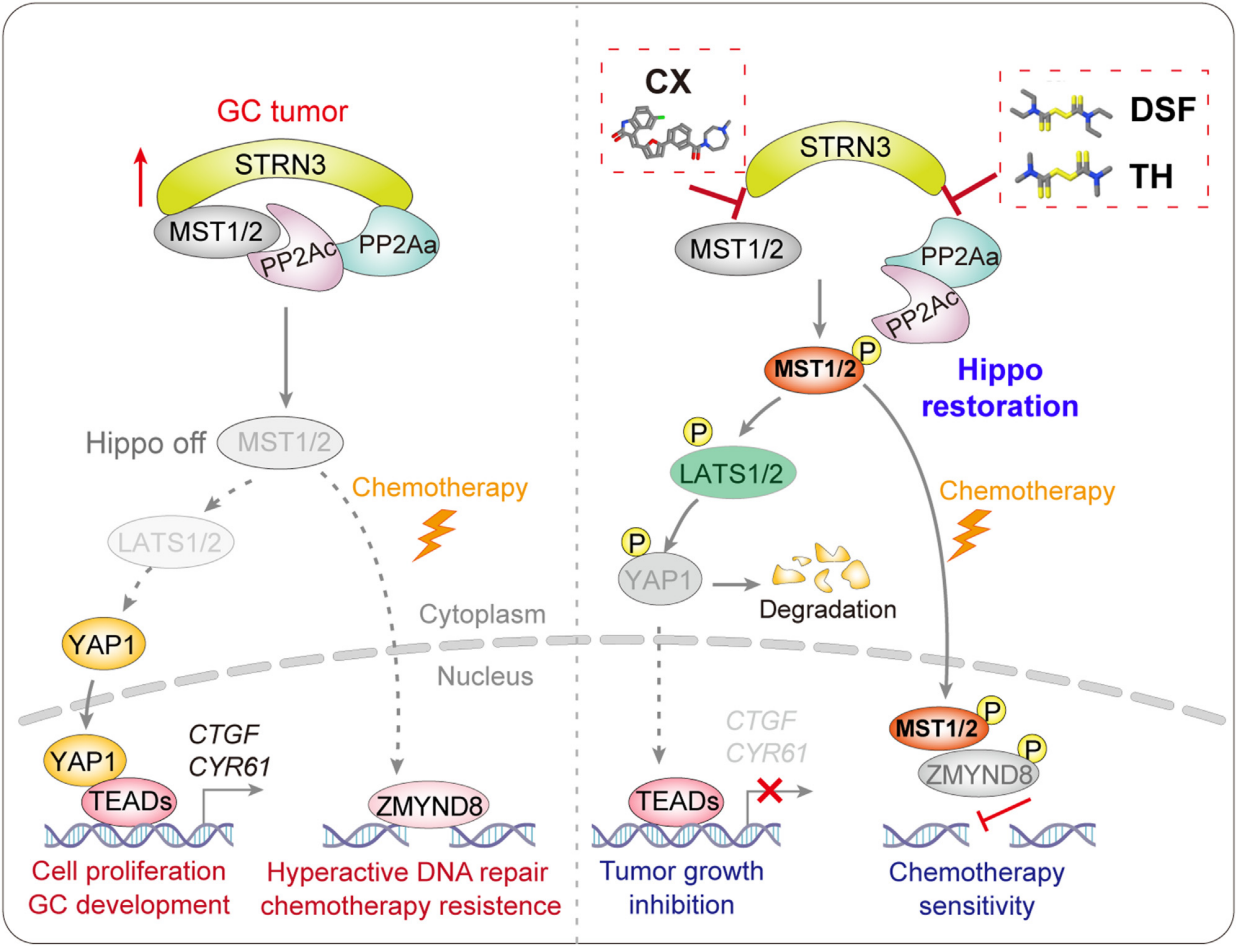
**A schematic illustration of the rationale of Hippo restoration for GC treatment.** The STRN3 acts as a bridge to link PP2A phosphatase and MST1/2 kinases, mediating the dephosphorylation of MST1/2. As such, the activity of MST1/2 is properly controlled. In tumors, STRN3 upregulation leads to enhanced inhibition of MST1/2 (Hippo off), resulting in the defective of LATS1/2-mediaed YAP dephosphorylation, transactivation of TEAD-dependent gene transcription, and GC development. Meanwhile, such sequestration of MST1/2 in the cytoplasm leads to elevated DNA repair capacity and drug resistance in response to chemotherapy (*left panel*). The FDA-approved drugs such as DSF and its structural analog TH, as well as CX, were concluded from the current work enabling to either block the PP2Aa–STRN3 or STRN3–MST1/2 interaction, respectively, and therefore restore the activity of MST1/2 (Hippo restoration) and in this way serve as a promising therapy for GC (*right panel*). CX, CX-6258; DSF, disulfiram; GC, gastric cancer.

The last 2 decades have witnessed great drug discovery effort towards the Hippo pathway against various diseases including tumors (5, 50, 51). Multiple candidates directly targeting YAP, TEADs, and their interactions have been identified. Our previous study identified STRN3 as a key regulatory subunit recruiting MST1/2 kinases to the PP2A core enzyme and thereby causing loss of Hippo tumor suppressor signaling in GC (16). The PP2A phosphatase has been characterized with multifaceted biological functions depending on the specific type of its regulatory subunit that is responsible for substrate recruitment. For example, PP2A phosphatase containing B55/56 regulatory subunit acts as a tumor suppressor by limiting the oncogenic activity of Myc in normal cells (52, 53) but may shift to play an oncogenic role due to upregulation of the STRN3 regulatory subunit in cancer cells (16). Thus, selective targeting of PP2A represents a long-standing challenge in the field of drug discovery. Previously, we showed that peptide mimetics-mediated selective targeting of the STRN3-containing PP2A can restore the Hippo signaling against cancer and that targeting STRIPAK complex can inhibit DNA repair therefore sensitizing cancer to PARP inhibition (16, 26). Here, we revisited the STRN3-containing PP2A and STRIPAK complex and discovered that pharmacological targeting of the PP2Aa–STRN3 or STRN3–MST1/2 interactions with FDA-approved drugs can potently recover the Hippo tumor suppressor signaling and selectively kill cancer cells (Fig. 7).

Our current work first identified the drug compound DSF that can disrupt the PP2Aa–STRN3 interaction to reactivate MST1/2 kinases for GC treatment. Previous studies have reported DSF as an anti-alcoholism drug and now indicated as an anticancer drug in a range of solid and hematological malignancies (54, 55, 56, 57). For example, DSF can inhibit cancer growth by inhibition of the p97–NPL4–UFD1 complex controlling protein homeostasis, as well as several other cellular pathways including ROS, PIK, MAPK, NF-κB, ALDH, EGFR/Src/VEGF (41, 58, 59, 60). Given its easy availability, cost effectiveness, and less adverse effects, DSF represents a highly promising anticancer drug candidate.

Our study also identified TH as a structural analog of DSF to exert similar function in terms of targeting the PP2Aa–STRN3 interactions and restores the Hippo signaling against cancer. Interestingly, TH has been reported as an inhibitor of angiogenesis and/or inflammation with NF-κB pathway activation (61) able to inhibit DNA synthesis and induce apoptosis in cultured bovine capillary endothelial cells (62, 63). Here, we repurposed DSF and TH for GC therapy and unexpectedly found that copper ion markedly enhanced the antitumor efficacy of both drugs, raising the possibility of combined pharmacotherapy of metal ion and small molecule drugs against GC. Moreover, given the similar molecular backbone shared by DSF and TH, it is a promising direction for further optimization of this line of compounds to gain better draggability and efficacy in the future (Fig. 7). Notably, some structural analogs of DSF such as CAS 5675-76-3 were found to also have significant antitumor activity, suggesting the potential to optimize the potency and selectivity of targeting the STRN3–PP2Aa interface (Fig. S5*B*).

Unlike kinases that are popular drug targets approved by the FDA, phosphatases have been long thought as undruggable due to their wide spectrum of substrates (64). Different holophosphatases are usually formed by the binding of the same catalytic core enzyme to distinct regulatory subunits. Therefore, currently available inhibitors targeting the catalytic activity of phosphatases are highly toxic and nonselective. Our previous structural characterization of the STRN3 interaction with PP2Aa uncovered a binding mode distinct from those of all known regulatory subunits including B, B′, and B″ types. This unique binding mode offers a special opportunity to have a very specific inhibitor design—with a new strategy involving the selective blocking of the recruitment or access of certain substrates to the PP2A core enzyme, avoiding most of the side effects from targeting the catalytic activity. Due to the good separation of the STRN3-binding site from the B/B′/B″-binding sites on PP2Aa, targeting the PP2Aa–STRN3 interface would not affect the binding of all other known regulatory subunits, therefore further excluding side effects caused by general intervention of broad substrate recruitment. Here, we took advantage of these insights to screen available drugs able to selectively target PP2A to unleash the suppressed Hippo signaling. Our rediscovery of the FDA-approved drug DSF exemplified such type of targeting strategy. Moreover, we also provide proof-of-concept for targeting the STRN3–MST1/2 interaction to reactivate Hippo against cancer.

Notably, previous studies have shown that DSF treatment can induce the reduction of body weight and glucose excretion in mice, suggesting *in vivo* side effects (65). In addition, DSF and TH also could cause adverse effects in zebrafish larvae (65, 66). Thus, future preclinical studies are warranted to translate our findings towards clinical trials, especially regarding pharmacokinetic, pharmacodynamic, and toxicity studies in animals. Moreover, further work is required to evaluate the efficacy of combinatorial treatment (*e.g.*, DSF + cisplatin) in different subtypes of tumors, such as STRN3 low-expressed and high-expressed GC.

In conclusion, our current work demonstrated a selective targeting of PP2Aa-STRN3 or STRN3-MST1/2 by the FDA-approved drugs to restore and even enhance the Hippo signal, which not only directly inhibit GC growth but also sensitize GC to chemotherapy.

## Experimental procedures

### Cell lines

HGC-27, AGS, and MGC-803 cells were obtained from the cell library of Chinese Academy of Sciences. NUGC-4 and MKN-45 were obtained from the cell bank of the RIKEN BioResource Center. MKN-28 was obtained from the JCRB Cell Bank (NIBIOHN). SNU-216 cells were obtained from the Korean Cell Line Bank (SNU). GES-1 and HEK293FT cells were obtained from Biofeng company. Insect cell lines (Sf9 and Hi-Five) were kind gifts from Prof. Jianping Ding (SIBCB). All human gastric cells were grown in RPMI 1640 (Invitrogen/Thermo Fisher Scientific). HEK293FT cells were grown in Dulbecco’s modified Eagle’s medium (Invitrogen/Thermo Fisher Scientific). Cell lines were maintained in culture supplemented with 10% heat-inactivated fetal bovine serum (Biological Industries) and 1% penicillin/streptomycin (Gibco/Thermo Fisher Scientific) at 37 °C with 5% CO_2_ in a humidified incubator (Thermo Fisher Scientific). Insect cells were grown in ESF 921 insect medium (Expression System) with 10% fetal bovine serum and 1% penicillin/streptomycin at 27 °C in 1 l glass Fernbach flasks with shaking at 100 rpm. All of the cells were passaged for 3 months from the frozen early-passage stocks that had been received from the indicated sources. During the study, all cell cultures were periodically tested for *mycoplasma* using MycoAlert *Mycoplasma* Detection Kits (Lonza).

### Mouse

The mice used in this study were from SLAC Laboratory Animal. All animals were housed under specific pathogen-free conditions in automated watered and ventilated cages on a 12 h light/dark cycle and handled in accordance with the guidelines of the Institutional Animal Care and Use Committee of the Shanghai Tenth People's Hospital, Tongji University. The approval ID for animals breeding was SHDSYY-2023-P0011.

### Plasmids

His-SUMO-tagged STRN3 (amino acid 64–145), His-tagged MST2 (amino acid 1–491), GST-tagged MST2 (amino acid 1–308), MBP-tagged MST2 (amino acid 1–308), and the mutant MBP-tagged MST2 (amino acid 1–308, L33/E35/V41A) were cloned and expressed in pET28a vectors as previously described. GST-tagged PP2Aa (amino acid 8–589) and its mutants were cloned and expressed in a pGEX-4T-1 vector. His-tagged PP2Ac (amino acid 1–309) was cloned and expressed in a pFastBac-HTA vector. For mammalian, cell-mediated transient expression constructs, including the Flag-tagged STRN3, Myc-tagged STRN3, Flag-tagged MST2, HA-tagged PP2Aa, and Flag-tagged YAP, as well as their related mutants, were sub-cloned into a modified pCDNA-3.1 vector.

### Protein purification

His-PP2A Cα (amino acid 1–309) proteins were expressed using the Bac-to-Bac Baculovirus expression system (Gibco) in *Spodoptera frugiperda* (Sf9) insect cells. The purification step was carried out as previously described (16). Other recombinant proteins used in this study were all expressed in *Escherichia coli* strain BL21 Coden plus (DE3) strain. The cells were grown at 37 °C in TB medium to the optical density of a sample at a wavelength of 600 nm (OD600) of 0.6 to 1.0, and the protein expression was induced with 0.25 mM IPTG at 18 °C for 18 h. The cells were harvested and suspended in a lysis buffer (20 mM Hepes, pH 7.5, 500 mM NaCl, 10% glycerol, and 1 mM DTT) and lysed with a high-pressure homogenizer. The lysates were centrifuged at 18,000 rpm for 30 min at 4 °C, and the supernatants were collected for further purification. For His-, His-sumo-, GST-, or MBP-tagged proteins, GST beads (GE Healthcare), His-beads, or MBP beads (Smart-lifesciences) were used for affinity chromatography according to the manufacturer’s instructions. The proteins were finally purified with size-exclusion chromatography in a buffer containing 20 mM Hepes, pH 7.5 and 100 mM NaCl.

### Pull-down assay

GST- and MBP-fused proteins were immobilized on glutathione agarose resin (NEB) and Dextrin Beads (Smart-lifesciences), respectively. After 2 h incubation with different prey proteins in a binding buffer containing 20 mM Hepes, pH 7.5, 100 mM NaCl at 4 °C, the beads were washed three times with binding buffer and were eluted with the same buffer plus 10 mM maltose monohydrate or 10 mM glutathione (adjusted to pH 8.0) (Sigma). The immobilized proteins were visualized with either Coomassie blue staining or Western blotting.

### MST assay

MST assay was performed using a Monoth NT.115 instrument from Nanotemper at 25 °C as described previously. Fluorescent-labeled proteins were then incubated with DSF or TH for 5 min before filling it into the capillaries. Fill 16 capillaries with the 16 mixes by dipping the capillaries into the sample, place the capillaries onto the capillary tray, and start the measurement. Data were analyzed using the MST analysis software version 2.3 (MO. Affinity Analysis, https://support.nanotempertech.com/hc/en-us/sections/17715198724753-Software).

### ITC assay

ITC experiments were conducted using an iTC200 instrument from Microcal at 25 °C. For calorimetric measurements, purified PP2Aa (8–589) or STRN3 (64–145) were loaded into the ITC cell at a concentration of 100 μM, and DSF in concentrations of 1 mM were auto-loaded into the syringe. All samples were in the same buffer containing 20 mM Hepes, pH 7.5, 100 mM NaCl. Each titration included a single 0.4 ml injection followed by 19 sequential injections of 2 ml aliquots, with a spacing of 300 s between the injections, and stirring at 1000 rpm. Data were analyzed using the ITC data analysis software (MicroCal Software, https://www.malvernpanalytical.com/en/support/product-support/software/microcal-peaq-itc-analysis-software-v141).

### *In vitro* dephosphorylation assay

The *in vitro* dephosphorylation system was used as previously described (16). Small molecules DSF and TH of indicated concentrations were mixed with autophosphorylated MST2 (2 mM) and in 20 ml of dephosphorylation buffer (20 mM Hepes pH7.5, 100 mM NaCl, 10 mM MgCl_2_, 1 mM DTT, Cocktail). After incubation at 30 °C for 30 min, the residual phosphorylation levels of MST2 were determined using a pMST1 (T183)/MST2 (T180) antibody (#3681, Cell Signaling Technology, 1:500).

### Real-time qPCR and RNA-Seq

For RT-PCR, total RNA was isolated from cells with RNA Isolater Total RNA Extraction Reagent (Vazyme Biotech) according to the manufacturer’s instructions. The isolated RNA was reverse transcribed into complementary DNA using HiScript II (Vazyme Biotech). Quantitative real-time PCR was performed with a CFX96 Real-Time system (Bio-Rad) and SYBR Green PCR master mix (Yeasen). The fold change in the gene expression was calculated using the comparative Ct method, and three replicates were tested for each complementary DNA sample. ACTB or Actb were used as an internal reference. Primers used in this study were as follows:

*hCTGF* forward: 5′-AAAAGTGCATCCGTACTCCCA-3′,

*hCTGF* reverse: 5′-CCGTCGGTACATACTCCACAG-3′;

*hCYR61* forward: 5′-GGTCAAAGTTACCGGGCAGT-3′,

*hCYR61* reverse: 5′-GGAGGCATCGAATCCCAGC-3′;

*hACTB* forward: 5′-ATCATGAAGTGTGACGTGGA-3′;

*hACTB* reverse: 5′-CTCAGGAGGAGCAATGATCT-3′.

For RNA-seq, HGC-27 cells in 6-well plates were treated with DSF, TH, or CX. Total RNA was extracted. RNA quality was assessed using a 2100 Expert Bioanalyzer (Agilent) and sent for library preparation and sequencing using the Illumina Hiseq2000 platform of Majorbio Biotech. The data were analyzed on the free online Majorbio I-Sanger Cloud Platform (www.majorbio.com). The GEO accession numbers for the high-throughput sequencing reported in this paper is GSE168618.

### Double strand break reporter assay

HEK293A cells were treated with DSF/DMSO for 24 h. Cells were continued to electroporated with the I-SceI expression construct (pCBASce) together with DR-GFP or EJ5-GFP reporter plasmid at 150 V, 975 μF using NEPA21 Super Electroporator (NEPA GENE). Cells were further recovered for 48 h after electroporation and were subjected for flow cytometric analysis using a BD FACS CantoII Analyzer.

### Immunoblotting

Whole cell extracts were prepared using lysis buffer (1% Triton-X 100, 10 mM EDTA, 1 mM PMSF, 1% Cocktail, pH = 7.4) and then boiled with SDS loading buffer and resolved in SDS-PAGE gels. The proteins were transferred to 0.45 mm polyvinylidene fluoride membranes (Bio-Rad) and were further incubated with the indicated antibodies. The antibodies used and their dilutions were listed as below: MST2 (ab52641, Abcam, 1:1000), Phospho-MST1(Thr183)/MST2(Thr180) (#3681, Cell Signaling Technology, 1:500), YAP(#14074, Cell Signaling Technology, 1:1000), p-YAP (S127) (#13008, Cell Signaling Technology, 1:1000), MOB1(#13730, Cell Signaling Technology, 1:1000), p-MOB1(T35) (#8699, Cell Signaling Technology, 1:1000), anti-HA (H3663, Sigma, 1:2000), anti-Flag (F3165, Sigma, 1:2000), β-actin (#A5441, Sigma, 1:5000), γ-H2AX (ab229914, Abcam, 1:2000), Phospho-Ser Antibody (sc-81514, Santa Cruz, 1:1000), Phospho-Tyr antibody (ab9332, Abcam, 1:1000). The secondary HRP antibodies including goat anti-rabbit (31460, 1:4000) and goat anti-mouse (31430, 1:4000) were purchased from Thermo Fisher Scientific. Western blotting images were captured by Mini Chemiluminescent Imaging and Analysis System (Beijing Sage Creation Science Co, LTD). Western blotting images were captured by using the Mini Chemiluminescent Imaging and Analysis System (Beijing Sage Creation Science).

### Cell viability and clonogenic assays

For cell viability assay, cells were seeded into 96-well format with 3000 cells per well. After attachment, cells were treated with indicated compounds (DSF or TH with/without Cu ion). Then an ATP-based CellTiter-Lumi Plus kit (Beyotime) was used according to the manufacturer’s instructions. The intracellular ATP contents were measured using a BioTek Synergy NEO multi-detector microplate reader (Thermo Fisher Scientific).

For the colony formation assay, HGC-27 cells treated with indicated compounds (DSF or TH) were seeded into 6-well format (600 cells per well). After about 2 weeks incubation, cell colonies were identified by their being stained with 1% crystal violet dye. And colonies with diameters of >1 mm were counted.

### MNNG-induced GC mouse model

The carcinogen-induced GC mouse model assay was approved by the Institutional Animal Care and Use Committee of Fudan University. Briefly, C57BL/6Slac mice were housed in an air-conditioned biohazard room designed for infectious animals, with a 12 h light: 12 h dark cycle. The GC mice model was created following a previously established protocol (23, 44). For each cycle, drinking water containing MNNG (100 mg/ml) was served for the mice for 14 consecutive days, and then normal drinking water was served for next 14 days. Three cycles of treatment were carried out to establish GC mice model. After 90 days of treatment, mice were scarified for subsequent analysis. The tumor size per stomach was counted. Mice were randomized to receive 50 mg/kg per day of DSF (HY-B0240, MCE) (n = 10 mice/group).

### Xenograft tumor model

The Xenograft tumor evaluation assay was approved by the Institutional Animal Care and Use Committee of Fudan University. Briefly, healthy male 615 mice were maintained in pathogen-free conditions. Mice were first injected with 2 × 10^6^ AGS cells/per mouse into their flanks to induce tumor formation. Once the tumor volume reached about 100 mm^3^, mice were injected intratumorally with indicted inhibitors for five successive days. Two weeks later, the mice were euthanized and then their tumors were harvested and weighed.

## Data availability

All the RNA-Seq sequencing data have been deposited in the Gene Expression Omnibus database under the accession no. GSE168618.

## Supporting information

This article contains supporting information.

## Conflict of interest

The authors have filed a patent (202110534762.X) for the thiram compound in China.
