# Supplementary material for: Reactivating Hippo by drug compounds to suppress gastric cancer and enhance chemotherapy sensitivity
Source: J Biol Chem. 2024 Apr 22;300(6):107311. doi: 10.1016/j.jbc.2024.107311 (PMC11126936; doi:10.1016/j.jbc.2024.107311)
Supplement: Supporting information [file mmc1.pdf]

SUPPLEMENTAL INFORMATION

Reactivating Hippo by Drug Compounds to Suppress Gastric Cancer and Enhance Chemotherapy Sensitivity

Zhifa Cao, Yu Hou, Zhangting Zhao, Hui Zhang, Luyang Tian, Yiming Zhang, Chao Dong, Fenghua Guo, Lijie Tan, Yi Han, Wenjia Wang, Shi Jiao, Yang Tang, Liwei An, Zhaocai Zhou

Supplementary Figures

Fig.S1. Illustration of the AlphaScreen and inhibition of 9 compounds in HGC-27 cells.
Fig.S2. DSF binds to PP2Aa to disrupt STRN3-PP2Aa interaction.
Fig.S3. DSF reactivates Hippo pathway to inhibit GC growth.
Fig.S4. Combined tumor-killing potency of cisplatin and DSF.
Fig.S5. TH functions as a structural analog of DSF for GC therapy.
Fig.S6. Identification of a STRN3-MST2 disruptor CX for Hippo-based GC therapy.

Supplementary Tables

Table S1. Primary AlphaScreen results for STRN3-PP2Aa disruption.
Table S2. Result of the 2nd round validation screen for STRN3-PP2Aa disruption.
Table S3. Primary AlphaScreen results for STRN3-MST2 disruption.
Table S4. Result of the 2nd round validation screen for STRN3-MST2 disruption.

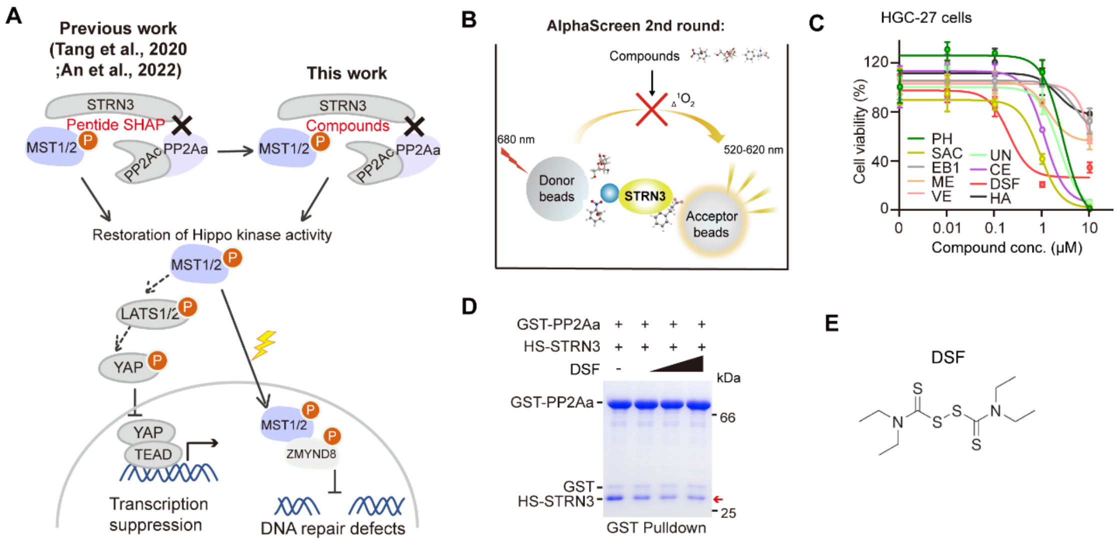

Fig.S1. Illustration of AlphaScreen and inhibition of 9 compounds in HGC-27 cells

A. Graphic illustration of our previous strategy for restoration of Hippo activity for cancer therapy by peptide inhibitor (SHAP) (previous work) or novel chemical compounds via disrupting PP2Aa-STRN3-MST1/2 interaction (this work). **B.** Illustration of the second-round AlphaScreen exclusion of false positive ones interrupting beads and protein association. **C.** Cell viability of HGC-27 cells treated with 9 indicated compounds. **D.** GST pulldown assay to validate the dose-dependent effect of DSF on disrupting the PP2Aa-STRN3 interaction. **E.** The chemical structure of DSF.

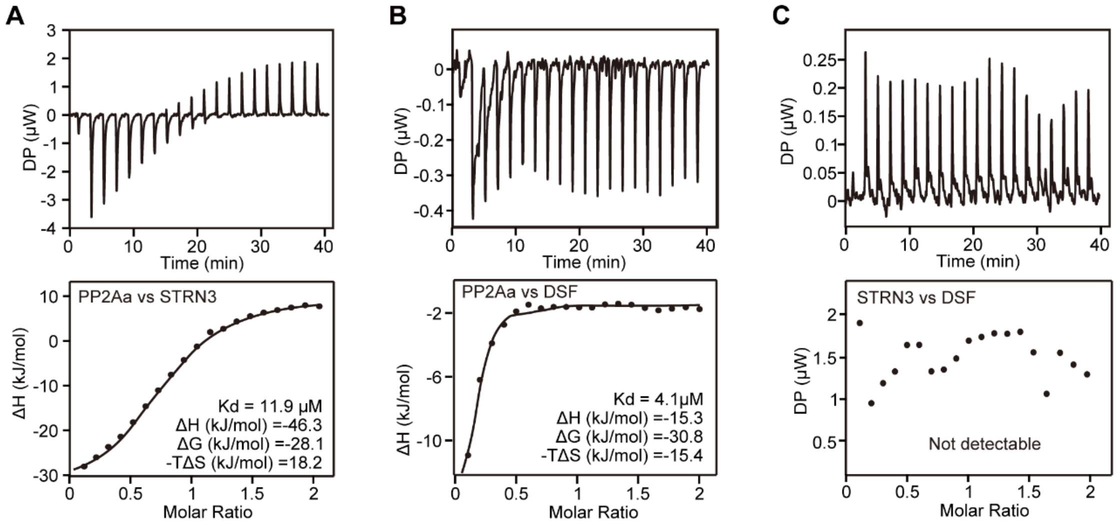

Fig.S2. DSF binds to PP2Aa to disrupt STRN3-PP2Aa interaction.

A. Detection of PP2Aa binding with STRN3 by Isothermal titration calorimetry (ITC) assay.

B. Detection of DSF binding with PP2Aa by ITC assay.

C. Detection the binding ability of DSF with STRN3 by ITC assay.

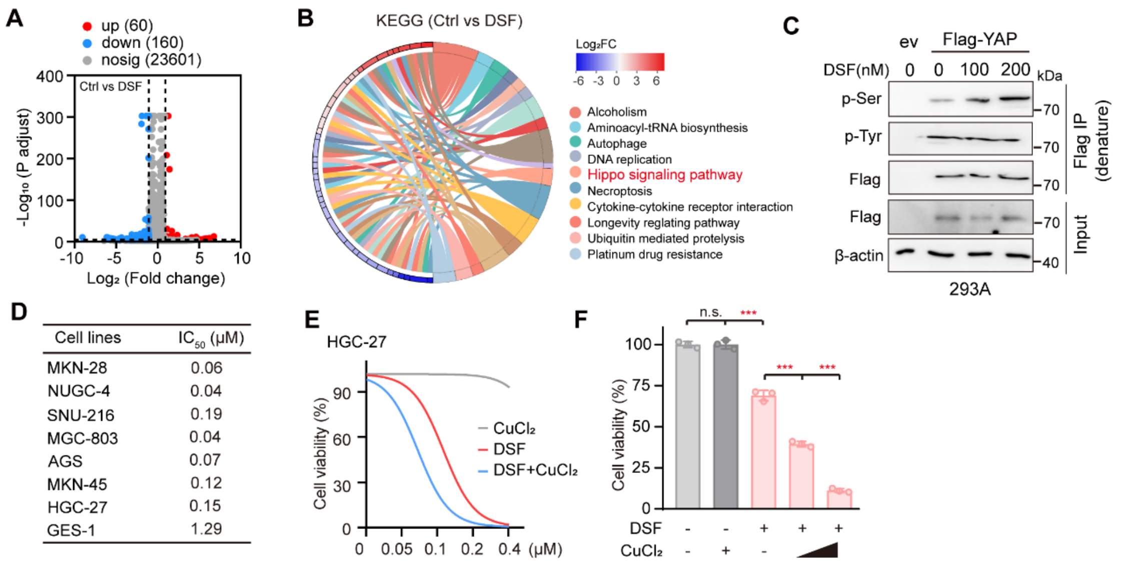

Cell lines	IC_{50} (μM)
MKN-28	0.06
NUGC-4	0.04
SNU-216	0.19
MGC-803	0.04
AGS	0.07
MKN-45	0.12
HGC-27	0.15
GES-1	1.29

Fig.S3. DSF reactivates Hippo to inhibit GC growth.

A. Volcano plot of altered genes in HGC-27 cells treated with DSF. **B.** KEGG enrichment analysis of altered pathways in DSF treated HGC-27 cells. **C.** Analysis of YAP Ser or Tyr phosphorylation upon DSF treatment in HEK293A cells transfected with Flag-YAP plasmids. **D.** IC_{50} values of 7 different GC cells lines (MKN-28, NUGC-4, SNU-216, MGC-803, AGS, MKN-45 and HGC-27) and 1 non-cancerous gastric epithelial cell line GES-1 towards DSF treatment. **E.** Cell viability of HGC-27 cells treated with DSF or DSF-Cu complex ($n = 3$). **F**. Cell viability of HGC-27 cells treated with DSF or DSF-Cu complex ($n = 3$). Data are presented as means ± SD. The data were analyzed using one-way ANOVA, followed by the Tukey's post-hoc test. ***, $p < 0.001$; n.s., no significance.

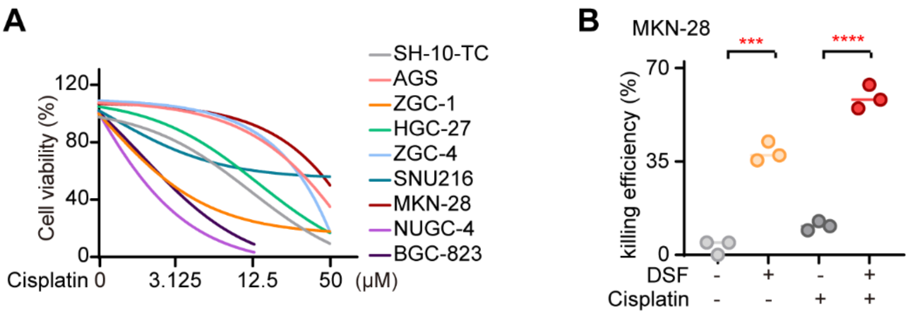

Fig.S4. Tumor-killing efficiency of combined treatment with cisplatin and DSF.

A. Cell viability of several indicated GC cell lines treated with cisplatin. **B.** Killing efficiencies of MKN-28 cells treated with DSF and cisplatin or in combination. Significance was tested using one-way ANOVA, followed by the Tukey's post-hoc test. ***, $p<0.001$; ****, $p<0.0001$.

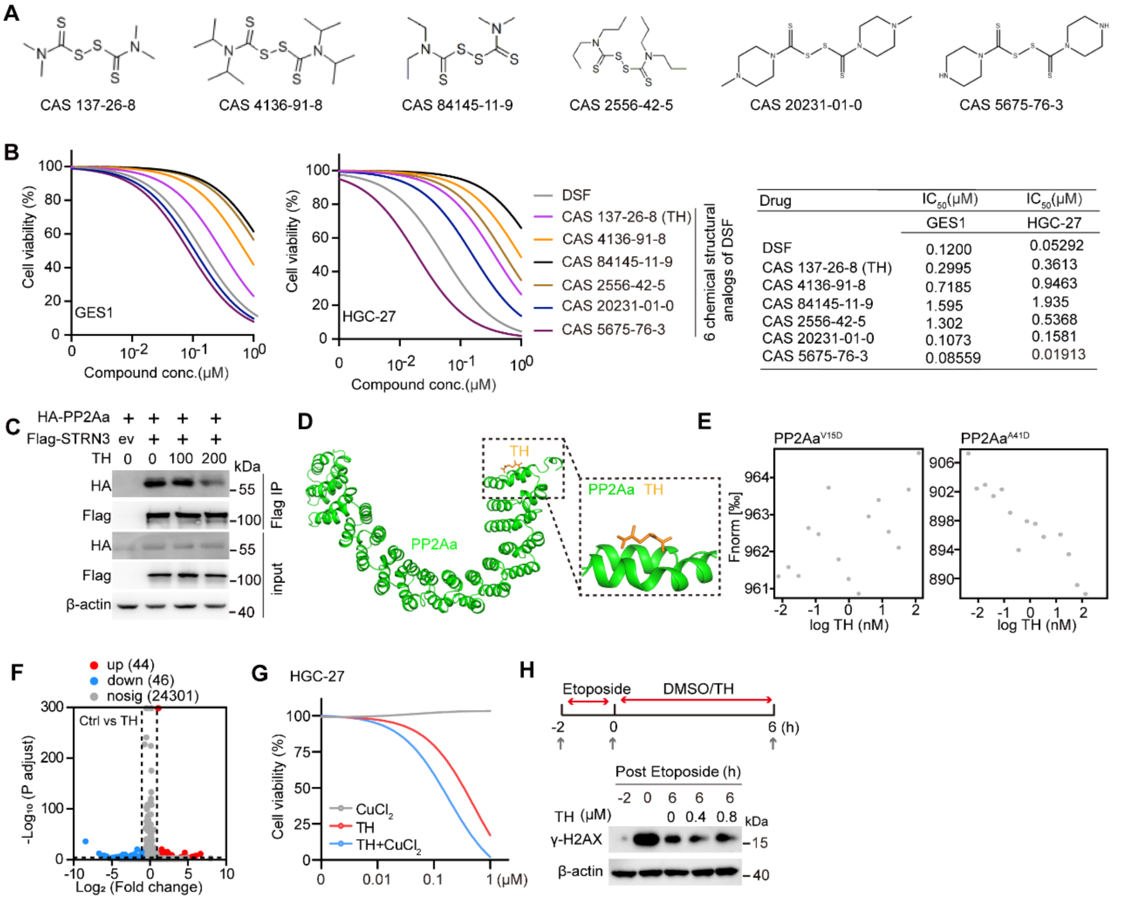

Drug	IC_{50}(μM) GES1	IC_{50}(μM) HGC-27
DSF	0.1200	0.05292
CAS 137-26-8 (TH)	0.2995	0.3613
CAS 4136-91-8	0.7185	0.9463
CAS 84145-11-9	1.595	1.935
CAS 2556-42-5	1.302	0.5368
CAS 20231-01-0	0.1073	0.1581
CAS 5675-76-3	0.08559	0.01913

Fig.S5. TH functions as a structural analog of DSF for GC therapy.

A. The top 6 chemical structural analogs of DSF with 90% similarity. **B.** Cell viability assay to assess the 6 DSF structural analogs in GES1 and HGC-27 cells. And their IC_{50} values were summarized on the right. **C.** Co-IP analysis of the interaction between PP2Aa and STRN3 in HEK293A cells treated with indicated doses of TH. **D.** Virtual docking of TH binding to first HEAT repeat of the PP2Aa. PP2Aa is colored in green and shown as cartoon. TH is shown as sticks and colored in yellow. The right panel illustrates the occupied position of TH in STRN3-PP2Aa complex. **E.** MST assay to show the incapability of PP2Aa single point mutants (V15D or A41D) in binding with TH. **F.** Volcano plot of altered genes in HGC-27 cells treated with TH. **G.** Cell viability of HGC-27 cells treated with $CuCl_2$, TH, TH-Copper complex (n = 3). **H.** Immunoblots of γ-H2AX expression incubated with or without TH. Experimental workflow of TH treatment in HEK293A cells pre-treated with 20 μM etoposide for 2 hr.

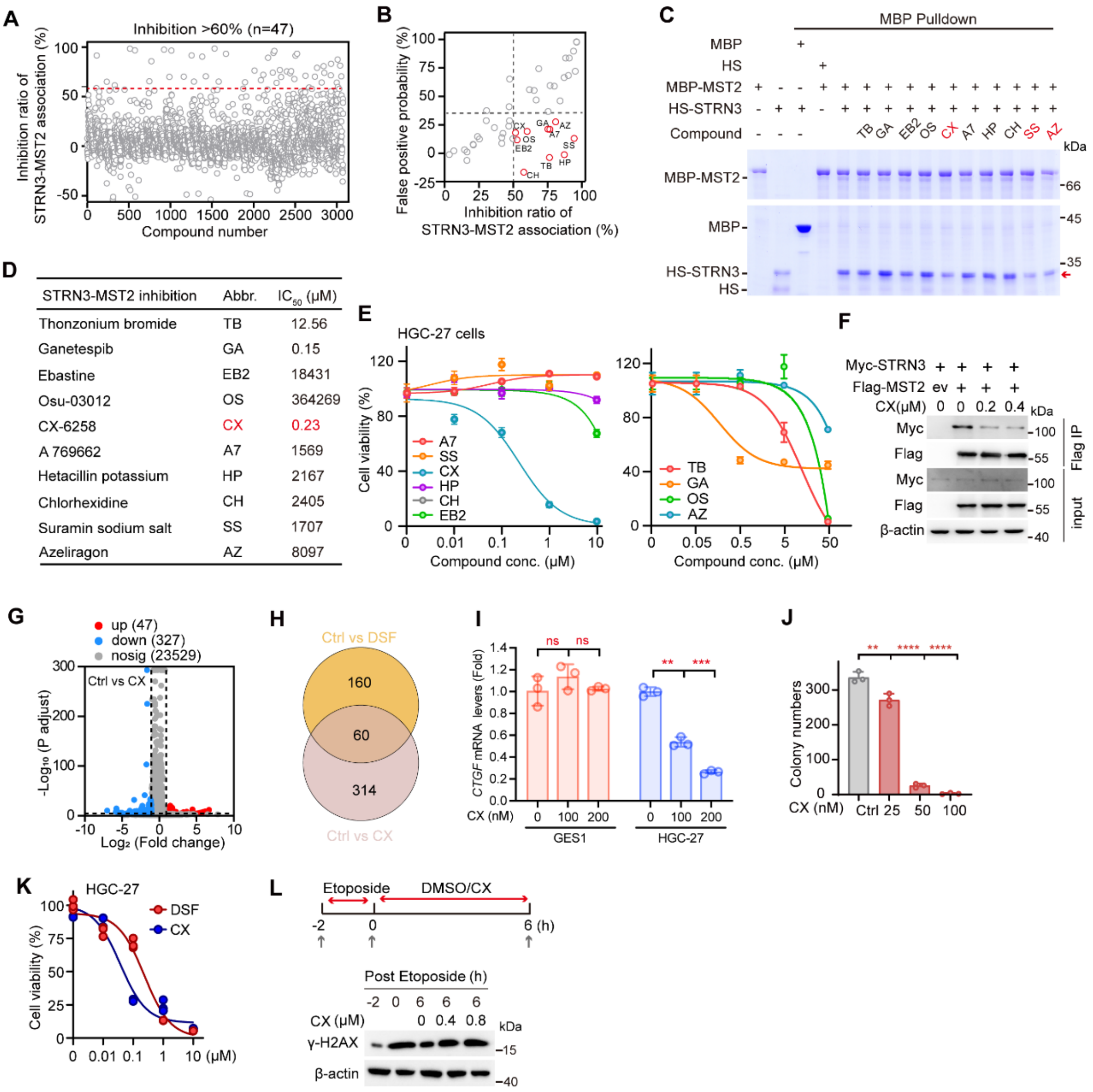

Fig.S6. Identification of a STRN3-MST2 disruptor CX for Hippo-based GC therapy.

A. Result of inhibition rate for disrupting the STRN3-MST2 interaction in the 1st round AlphaScreen. Dash line refers to inhibition efficiency higher than 60% (n=47). **B.** Result of the 2nd round AlphaScreen to kick out false positive hits which disrupted interactions between protein and donor/acceptor beads instead of breaking the STRN3-MST2 protein-protein interaction. Vertical dash line refers to inhibition efficiency higher than 50% while horizontal dash line refers to false positive probability less than 35% (n=10). **C.** MBP pulldown assay to assess the disruption effect of STRN3-MST2 association with the indicated 10 compounds treatment. HS: His-Sumo tag. **D.** IC_{50} values of the 10 candidate compounds in HGC-27 cells assessed by cell viability assay. **E.** Cell viability of HGC-27 cells treated with 10 indicated compounds. **F.** Co-IP analysis of the interaction between PP2Aa and STRN3 in HEK293A cells treated with or without CX. **G.** Volcano plot of altered

genes in HGC-27 cells treated with CX. **H.** Venn diagram illustration of altered genes in both gene sets treated with DSF and CX. **I.** Relative mRNA levels of *CTGF* versus the DMSO control in GES1 and HGC-27 cells treated with CX for 48 hr (n = 3). Data are presented as means ± SD. The data were analyzed using one-way ANOVA, followed by the Tukey's post-hoc test. **, $p<0.01$; ***, $p < 0.01$; n.s., no significance. **J.** Colony number of HGC-27 cells treated with CX corresponding to figure 7H. Significance was tested using one-way ANOVA, followed by the Tukey's post-hoc test. **, $p<0.01$; ****, $p<0.0001$. **K.** Cell viability of HGC-27 cells treated with DSF and CX respectively. **L.** Immunoblots of γ-H2AX expression incubated with or without CX. Experimental workflow of CX treatment in HEK293A cells pre-treated with 20 μM etoposide for 2 hr.
